# LDLRAD2 drives glycolysis and angiogenesis to promote extramedullary infiltration in acute myeloid leukemia

**DOI:** 10.1016/j.isci.2026.115987

**Published:** 2026-05-20

**Authors:** Kexin Jin, Yang Zhao, Qiang Guo, Jingyi Lin, Hongli Zhao, Shengjin Fan, Chuiming Jia, Shuchuan Liu, Desheng Kong

**Affiliations:** 1 Department of Hematology, The Fourth Affiliated Hospital of Harbin Medical University, Harbin 150006, China; 2 Department of Hematology, The First Affiliated Hospital of Harbin Medical University, Harbin 150001, China; 3 Hematology Department, Harbin Medical University Cancer Hospital, Harbin 150081, China

**Keywords:** Health sciences, Pathology, Biochemistry

## Abstract

Acute myeloid leukemia (AML) remodels the bone marrow microenvironment and can spread to extramedullary sites, a feature associated with adverse clinical outcomes. Here, we identify low-density lipoprotein receptor class A domain-containing 2 (LDLRAD2) as a driver of extramedullary infiltration (EMI) through analyses of patient samples, public datasets, AML cell models, and xenografts. LDLRAD2 increased glucose consumption, lactate production, endothelial tube formation, spleen infiltration, and microvascular density, whereas its knockdown reduced these phenotypes. Mechanistically, LDLRAD2 interacted with metadherin (MTDH) and activated PI3K/AKT/mTOR signaling, thereby promoting glycolysis, angiogenesis, and EMI. Inhibition of LDLRAD2 or treatment with 2-deoxyglucose suppressed glycolysis-associated angiogenic effects. These findings support LDLRAD2-associated metabolic and angiogenic remodeling as a mechanism underlying EMI in AML and highlight a potential therapeutic vulnerability.

## Highlights

• LDLRAD2 is overexpressed in EMI-AML patients and correlates with shorter survival
• LDLRAD2 enhances glycolysis and lactate production to promote AML infiltration
• LDLRAD2 activates MTDH/PI3K/AKT/mTOR signaling to promote angiogenesis and EMI
• LDLRAD2 inhibition or 2-deoxyglucose suppresses EMI-associated angiogenesis

## Graphical abstract

**Figure:**
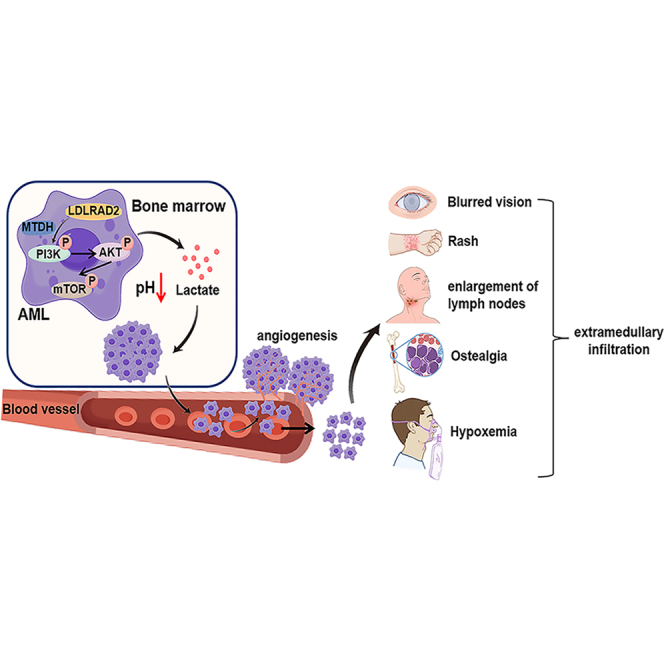

## Introduction

Acute myeloid leukemia (AML) is an aggressive hematological malignancy characterized by uncontrolled expansion, self-renewal capacity, and disrupted differentiation of clonal hematopoietic stem cells and progenitor cells.^1^ Epidemiological studies have revealed that patients younger than 60 years of age exhibit a modest 5-year survival rate of 35%–40%, whereas elderly and high-risk populations experience a grim prognosis, with reported survival rates of less than 15%.^2^^,^^3^ Recent advances in targeted therapies, including FLT3 inhibitors (midostaurin), IDH1/2 inhibitors (ivosidenib), and BCL-2 inhibitors (venetoclax), have markedly improved response rates in select patients.^4^ However, mortality rates exceed 60% in patients with relapsed or refractory AML.^5^ Consequently, a deeper understanding of AML pathogenesis and the development of novel therapeutic strategies remain critical priorities in contemporary hematological research.

AML cells infiltrate extramedullary tissues and organs beyond the bone marrow by releasing cytokines, adhesion molecules, and extracellular matrix components, which is clinically recognized as extramedullary infiltration (EMI).^6^^,^^7^^,^^8^ Approximately 30%–40% of newly diagnosed AML patients exhibit EMI, which manifests as lymphadenopathy, gingival hyperplasia, cutaneous lesions, hepatosplenomegaly, or central nervous system involvement.^9^ EMI in patients with AML significantly compromises treatment efficacy, which frequently leads to relapse and increased mortality rates.^10^ Given its adverse impact on relapse, elucidation of the molecular mechanisms of EMI, as well as the translation of these insights into clinically actionable biomarkers and targeted therapies, remains a critical unmet need in AML research.^11^

In AML cells, a preference for aerobic glycolysis (commonly known as the “Warburg effect”) persists, even under oxygen-sufficient conditions. This metabolic preference not only fuels rapid leukemic proliferation by supplying energy and biosynthetic precursors but may also contribute to disease progression by altering the bone marrow microenvironment.^12^ Intriguingly, research has demonstrated that glycolytic inhibitors can suppress AML cell growth, induce apoptosis, and increase chemosensitivity, thus implicating glycolysis as a key player in AML pathogenesis.^13^^,^^14^ However, the specific mechanisms underlying EMI remain unclear.

Research has demonstrated that the remodeling of the bone marrow microenvironment in AML is closely related to angiogenesis.^15^^,^^16^^,^^17^^,^^18^ Microvessel density (MVD), a key angiogenesis marker, is elevated in adult AML patients and correlates with disease aggressiveness and poor prognosis.^19^^,^^20^ AML cells exhibit a pronounced glycolytic phenotype, thus leading to significant lactate accumulation and hypoxia-inducible factor 1-alpha (HIF-1a) stabilization, which potently upregulates the expression of angiogenic mediators, particularly vascular endothelial growth factor (VEGF).^21^ These newly formed vessels not only nourish leukemic cells but also facilitate their infiltration into peripheral tissues. However, VEGF inhibitors have demonstrated limited efficacy, suggesting the involvement of alternative pathways.^22^^,^^23^ Thus, investigation of glycolysis and angiogenesis in AML may reveal disease mechanisms and offer new strategies to combat EMI-AML.

The low-density lipoprotein receptor class A domain-containing 2 (*LDLRAD2*) gene encodes a transmembrane protein implicated in tumor progression. Elevated LDLRAD2 expression has been observed in multiple malignancies such as diffuse large B cell lymphoma (DLBCL) and pancreatic cancer (PC), which is correlated with increased cell proliferation, invasiveness, and poor prognosis.^24^ In gastric cancer (GC), LDLRAD2 facilitates tumor migration, invasion, and metastatic dissemination by activating the Wnt/β-catenin signaling cascade.^25^ However, the functional significance and underlying molecular mechanisms of LDLRAD2 in AML remain poorly understood.

In this study, we observed higher expression levels of LDLRAD2 in AML patients with EMI than in those without EMI. This increased expression was characterized by an increased number of bone marrow vessels and increased glycolytic protein levels and was closely associated with poor prognosis. Functional assays revealed that LDLRAD2 enhances AML cell proliferation, glycolytic activity, and tube formation in human umbilical vein endothelial cells (HUVECs). Notably, treatment with LDLRAD2 shRNA or the glycolysis inhibitor 2-deoxyglucose (2-DG) attenuated angiogenic responses in AML. Mechanistically, LDLRAD2 may interact with metadherin (MTDH), thereby leading to the activation of the phosphoinositide 3-kinase (PI3K)/AKT/mTOR pathway, which correspondingly promotes glycolysis in AML cells, stimulates angiogenesis, and ultimately drives EMI. These findings highlight the targeting of LDLRAD2 or the use of glycolysis inhibitors as potential therapeutic options for EMI-AML.

## Results

### LDLRAD2 is highly expressed in EMI-AML patients and AML cell lines and predicts poor prognosis

**Figure 1:**
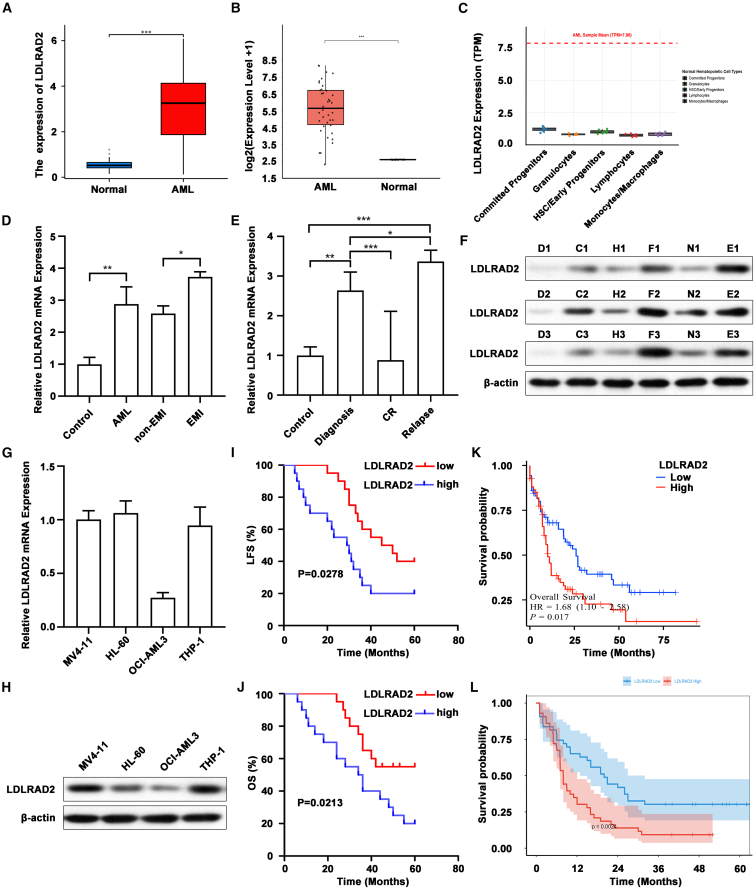
LDLRAD2 is highly expressed in EMI-AML patients and AML cell lines and predicts poor prognosis (A) Cross-database validation using GEPIA2 (integrating TCGA and GTEx data) confirmed elevated *LDLRAD2* expression in AML (*n* = 173) compared to normal tissues (*n* = 70). (B) Independent validation in a TARGET AML subset with available expression data (AML *n* = 38) together with the GSE9476 dataset (normal *n* = 38). (C) Analysis of the GSE107011 dataset shows that *LDLRAD2* expression in AML does not follow the differentiation trajectory of 29 normal hematopoietic subsets (CD34^+^ HSCs, CMPs, GMPs, monocytes, and granulocytes), indicating leukemia-specific expression. (D) Compared with that in normal tissues (*n* = 10), *LDLRAD2* mRNA expression was higher in AML samples obtained at EMI (*n* = 20) than in matched AML samples obtained at non-EMI (*n* = 20). (E) RT-qPCR of BMMCs revealed higher *LDLRAD2* mRNA expression in samples obtained at diagnosis and relapse than in CR samples. Control, *n* = 10; diagnosis, *n* = 15; CR, *n* = 10; relapse, *n* = 15. (F) Western blot analysis of LDLRAD2 protein expression in clinical BMMC samples. (G) The expression of *LDLRAD2* mRNA in AML cell lines was measured by RT-qPCR. (H) Western blot analysis of LDLRAD2 protein expression in AML cell lines. (I and J) Kaplan-Meier analysis demonstrated that high *LDLRAD2* mRNA expression was associated with poorer LFS and OS in AML patients (*n* = 40). (K) Validation of *LDLRAD2* prognostic value in the TCGA-AML cohort (*n* = 173). (L) Independent validation of *LDLRAD2* prognostic value in a TARGET AML subset with complete OS information (*n* = 86). Unless otherwise indicated, data are presented as mean ± SD. A and B compare independent public cohort samples. D was analyzed using Welch’s two-tailed unpaired t tests. E was analyzed by one-way ANOVA followed by Tukey’s multiple-comparison test. Survival differences in I–L were analyzed using the Kaplan-Meier method with log rank test. *n* indicates biologically independent patient samples, public cohort samples, or independent biological experiments, as specified for each panel. ∗*p* < 0.05, ∗∗*p* < 0.01, and ∗∗∗*p* < 0.001. D1–D3: healthy controls, C1–C3: diagnosis, H1–H3: CR, F1–F3: relapse, N1–N3: non-EMI, E1–E3: EMI, EMI, extramedullary infiltration; CR, complete remission; LFS, leukemia-free survival; OS, overall survival.

To explore the molecular pathogenesis of EMI-AML, we initially performed a comparative transcriptomic analysis using the GSE116616 dataset, which included bone marrow samples from 8 AML patients (4 EMI and 4 non-EMI patients) and 4 healthy controls. The results demonstrated that *LDLRAD2* mRNA expression was significantly higher in AML samples than in normal controls (log2FC > 1, *p* < 0.05; Figure S1A). To validate our initial findings, we performed cross-database validation using GEPIA2, which integrates TCGA (173 AML patients) and GTEx (70 normal controls) data. The results consistently demonstrated significantly higher *LDLRAD2* expression in AML samples than in normal controls (*p* < 0.001; Figure 1A). We further validated these observations in an independent TARGET AML subset with available expression data (*n* = 38), together with the GSE9476 dataset (*n* = 38 normal controls), which also confirmed elevated *LDLRAD2* expression in AML (Figure 1B). To determine whether high *LDLRAD2* expression is leukemia-specific rather than a consequence of differentiation status, we analyzed its expression across 29 normal human hematopoietic populations using the GSE107011 dataset. *LDLRAD2* expression in AML was independent of differentiation status. Its levels did not align with the trajectory of normal CD34^+^ hematopoietic stem cells (HSCs), common myeloid progenitors (CMPs), granulocyte-monocyte progenitors (GMPs), or mature monocytes/granulocytes (Figure 1C). These findings indicate that elevated LDLRAD2 in AML is independent of cellular differentiation, confirming that its high expression is AML-specific.

To further corroborate these findings, we subsequently expanded our collection of clinical samples, including 40 AML patients (20 EMI-AML patients and 20 non-EMI-AML patients) and 10 healthy controls, to assess the expression levels of *LDLRAD2* mRNA and protein. The detailed clinical and laboratory parameters are presented in Table S1. Notably, both *LDLRAD2* mRNA expression and protein abundance, as assessed by Western blotting, were markedly elevated in EMI-AML samples compared with non-EMI-AML samples (Figures 1D and 1F; Figure S3A). Additionally, RT-qPCR and Western blot analysis of bone marrow mononuclear cells (BMMCs) revealed dynamic changes in *LDLRAD2* expression levels at different disease stages. Notably, the highest expression levels were observed at the initial diagnosis and relapse but were significantly decreased upon achieving CR (Figures 1E and 1F). Comparative evaluations of AML cell lines revealed cell type-specific expression profiles, with HL-60 cells exhibiting the highest LDLRAD2 levels and OCI-AML3 cells demonstrating minimal expression (Figures 1G and 1H; Figure S3B). To determine whether *LDLRAD2* expression correlated with specific AML genetic subtypes, we analyzed the GSE6891 dataset (537 AML patients with different *FLT3*, *IDH1*, and *NPM1* mutation subtypes). The results showed no statistically significant correlation between *LDLRAD2* expression and *FLT3* (*p* = 0.32), *IDH1* (*p* = 0.28), or *NPM1* (*p* = 0.07) mutation (Figure S1B), indicating that LDLRAD2 may act as an independent molecular marker in AML. Subsequently, survival analysis revealed significant clinical correlations, as 20 patients with elevated *LDLRAD2* expression had poorer LFS (Figure 1I, *p* = 0.0278) and OS (Figure 1J, *p* = 0.0213) than those with low *LDLRAD2* expression. This prognostic value was further validated in two independent cohorts, the TCGA-AML cohort (*n* = 173; *p* = 0.018; Figure 1K) and an independent TARGET AML subset with complete OS information (*n* = 86; *p* = 0.023; Figure 1L), underscoring the stability of LDLRAD2 as a prognostic biomarker in AML.

### EMI-AML patients are characterized by elevated LDLRAD2 expression, increased MVD, and increased glycolytic protein levels

**Figure 2:**
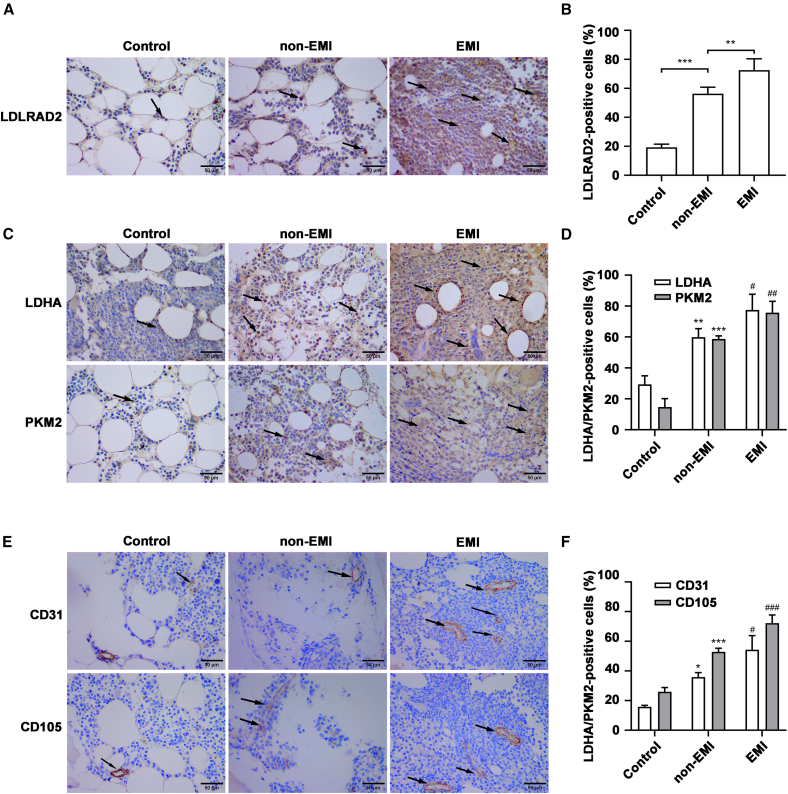
EMI-AML patients are characterized by elevated LDLRAD2 expression, increased MVD, and increased glycolytic protein levels (A) Representative IHC staining of bone marrow biopsy samples from AML patients revealed higher expression of LDLRAD2 in EMI-AML patients than in controls. (B) Quantitative analysis of the percentage of LDLRAD2-positive cells measured from IHC staining. (C) Representative IHC staining of the glycolysis-related proteins LDHA and PKM2 in bone marrow biopsy samples from AML patients and controls. (D) Quantitative analysis of the percentage of LDHA-positive and PKM2-positive cells measured from IHC staining. (E) Representative IHC staining of bone marrow biopsy samples from AML patients revealed increased MVD in EMI-AML patients than in controls. (F) Quantitative analysis of MVD, presented as both the percentage of CD31-positive and CD105-positive cells measured from IHC staining. The original magnification was 200×; scale bars, 50 μm. Data are presented as mean ± SD. Representative images are shown from one control specimen, one non-EMI specimen, and three EMI specimens. Quantification was performed from three representative fields per bone marrow biopsy specimen. B, D, and F were analyzed by one-way ANOVA followed by Tukey’s multiple-comparison test; for D and F, statistical analyses were performed separately for each marker. ∗*p* < 0.05, ∗∗*p* < 0.01 and ∗∗∗*p* < 0.001 versus control; ^#^*p* < 0.05, ^##^*p* < 0.01 and ^###^*p* < 0.001 versus non-EMI. Scale bars, 50 μm. Arrows indicate positive cells. EMI, extramedullary infiltration.

Emerging evidence suggests that aberrant glycolytic metabolism is a hallmark of AML and supports the rapid proliferation and survival of leukemic blasts.^26^^,^^27^^,^^28^ Key glycolytic enzymes, such as LDHA and PKM2, drive lactate production and anabolic precursor synthesis.^29^^,^^30^ To investigate this phenomenon in EMI-AML, we analyzed LDLRAD2, glycolytic markers (LDHA and PKM2), and MVD (CD31 and CD105) expression in bone marrow biopsies by IHC. We observed that compared with non-EMI-AML patients and healthy controls, EMI-AML patients had a significantly greater percentage of LDLRAD2-positive cells (Figures 2A and 2B). Similarly, the percentage of cells expressing the glycolytic enzymes LDHA and PKM2 markedly increased in the EMI-AML group (Figures 2C and 2D). Assessment of the MVD using the endothelial markers CD31 and CD105 revealed that EMI-AML bone marrow had both a greater percentage of CD31^+^ and CD105+ cells and an increased mean optical density of staining, indicating enhanced vascularity (Figures 2E and 2F). In summary, our findings indicate that LDLRAD2 expression positively correlated with the levels of glycolysis-related proteins (LDHA and PKM2), suggesting its potential involvement in the regulation of glycolytic reprogramming in EMI-AML.

### LDLRAD2 promotes proliferation and migration via glycolysis in AML cells

**Figure 3:**
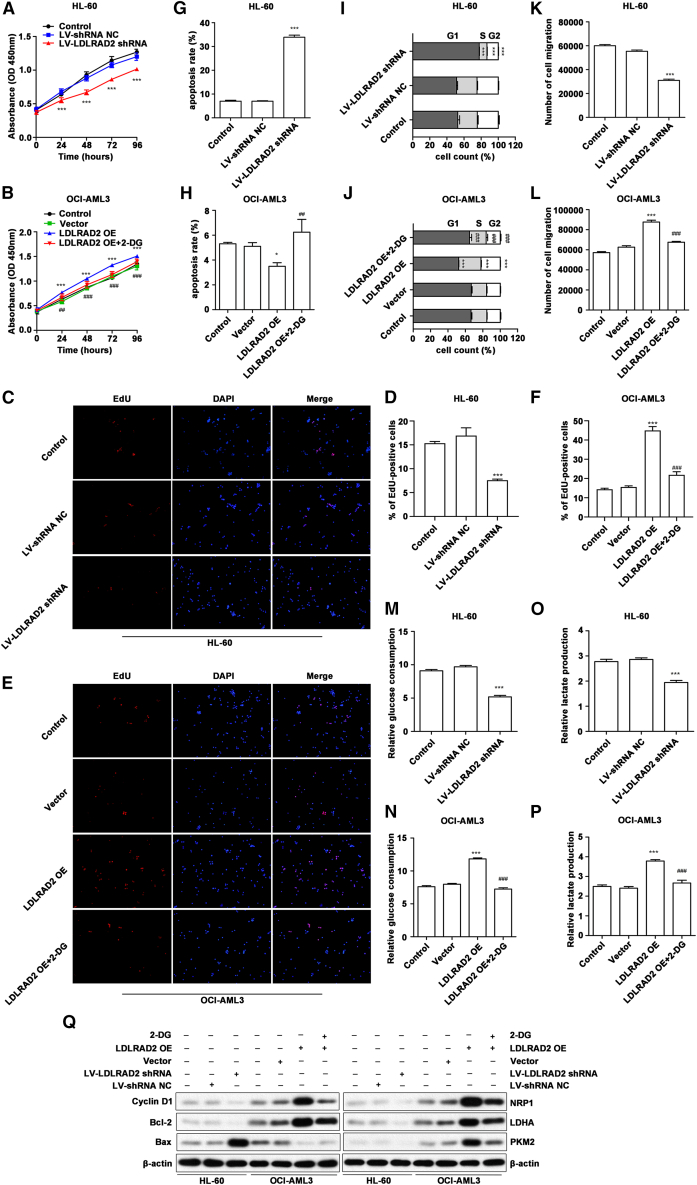
LDLRAD2 promotes proliferation and migration via glycolysis in AML cells (A) The knockdown of *LDLRAD2* significantly inhibited the proliferation of HL-60 cells. (B) The overexpression of *LDLRAD2* significantly enhanced the proliferation of OCI-AML3 cells. (C) The proliferation of HL-60 cells measured by EdU assay. The original magnification was 200×. (D) Quantification of the proliferation quantity of EdU-positive cells. (E) The proliferation of OCI-AML3 cells measured by EdU assay. The original magnification was 200×. (F) Quantification of the proliferation quantity of EdU-positive cells. (G) The knockdown of *LDLRAD2* increased the number of apoptotic cells of HL-60 cells. (H) The overexpression of *LDLRAD2* decreased apoptosis of OCI-AML3 cells. (I) The knockdown of *LDLRAD2* decreased the proportion of HL-60 cells in the S phase. (J) The overexpression of *LDLRAD2* increased the proportion of OCI-AML3 cells in the S phase. (K and L) Transwell migration assays demonstrated that *LDLRAD2* knockdown inhibited HL-60 cell migration, whereas *LDLRAD2* overexpression promoted OCI-AML3 cell migration. (M and N) Glucose consumption in HL-60 cells and OCI-AML3 cells. (O and P) Lactate production in HL-60 cells and OCI-AML3 cells. Nontransfected cells were used as a control, and the vector or shRNA NC was used as a negative control. (Q) Representative western blots showing the expression of Bcl2, Bax, cyclin D1, NRP1, LDHA, and PKM2 in AML cells. Nontransfected cells were used as a control, and β-actin was used as the loading control. Data are presented as mean ± SD from three independent biological experiments for quantitative assays. A and B were analyzed by two-way ANOVA followed by Bonferroni posttests. D, F, G, H, I, J, K, L, M, N, O, and P were analyzed by one-way ANOVA followed by Tukey’s multiple-comparison test. Q shows representative western blots. ∗*p* < 0.05, ∗∗*p* < 0.01 and ∗∗∗*p* < 0.001 versus control; ^#^*p* < 0.05, ^##^*p* < 0.01 and ^###^*p* < 0.001 versus the LDLRAD2 OE group. β-actin was used as the loading control. OE, overexpression; NC, negative control.

To investigate the tumor-promoting function of LDLRAD2 in AML and EMI, we first designed three shRNAs targeting *LDLRAD2* and a negative control shRNA (shNC). After validating their knockdown efficiency by qPCR and western blotting, we selected the most effective shRNA (Figures S1C–S1E). Subsequently, we established *LDLRAD2* knockdown in HL-60 cells using lentiviral shRNA delivery and transfected *LDLRAD2* overexpression plasmid vectors into OCI-AML3 cells (Figures S1F–S1H), thereby establishing the respective knockdown and overexpression cell models. Subsequent functional analyses revealed significant alterations in cellular behavior and metabolic activity. In the CCK-8 proliferation assays, *LDLRAD2* knockdown resulted in marked growth suppression (Figure 3A), whereas *LDLRAD2* overexpression enhanced the proliferation of OCI-AML3 cells (Figure 3B). Given that CCK-8 assays rely on dehydrogenases (metabolic enzymes) and may be affected by metabolic changes, we performed a direct, metabolism-independent proliferation assay (EdU incorporation assay) to measure DNA synthesis directly. The results corroborated the proliferation data: *LDLRAD2* knockdown significantly decreased the percentage of EdU-positive HL-60 cells, whereas *LDLRAD2* overexpression increased the percentage of OCI-AML3 cells (Figures 3C–3F). Flow cytometry analysis demonstrated that LV-*LDLRAD2* shRNA significantly induced apoptosis in HL-60 cells (Figure 3G; Figure S2A) and increased the percentage of cells in the G1 phase (from 52.7% to 77.7%; Figure 3I; Figure S2C). In contrast, *LDLRAD2*-overexpressing OCI-AML3 cells exhibited reduced apoptosis rates (Figure 3H; Figure S2B) and decreased the percentages of G1-phase cells (from 66.4% to 52.3%; Figure 3J; Figure S2D). Transwell migration assays revealed that *LDLRAD2* knockdown reduced HL-60 cell migration, whereas *LDLRAD2* overexpression increased the migration of OCI-AML3 cells (Figures 3K and 3L). Furthermore, overexpression of *LDLRAD2* in OCI-AML3 cells resulted in increased glucose consumption and lactate production compared to that in HL-60 cells (Figures 3M–3P).

To explore the underlying mechanisms of this effect, we assessed key regulatory proteins, including proteins related to cell cycle progression (Cyclin D1), apoptosis (Bcl-2 and Bax), migration (NRP1), and glycolytic metabolism (LDHA and PKM2) by western blotting. Notably, *LDLRAD2* knockdown downregulated the expression of cyclin D1, Bcl-2, NRP1, LDHA, and PKM2, while upregulating the expression of the proapoptotic factor Bax in AML cell lines (Figure 3Q; Figures S3C–S3D). These results indicated that LDLRAD2 promotes disease progression and is closely associated with glycolysis.

To determine whether the LDLRAD2-driven oncogenic effects are dependent on glycolysis, we treated *LDLRAD2*-overexpressing OCI-AML3 cells with 2-DG. 2-DG is a glucose analog that exerts antitumor effects by inhibiting glycolysis.^31^ This treatment counteracted LDLRAD2-driven proliferation and apoptosis resistance in OCI-AML3 cells while downregulating NRP1, BCL2, and cyclin D1 expression. The results of our study demonstrated that LDLRAD2 exerts oncogenic effects by modulating glycolytic pathways *in vitro* (Figure 3).

### LDLRAD2-derived supernatant enhances angiogenic tube formation in HUVECs

**Figure 4:**
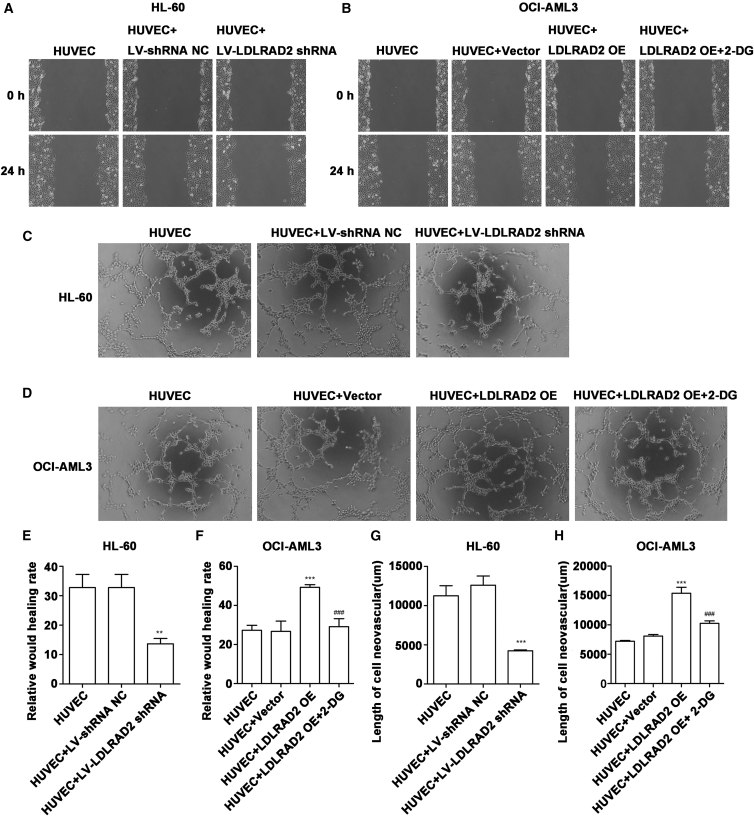
LDLRAD2-derived supernatant increases angiogenic tube formation in HUVECs (A) A wound healing assay revealed inhibited HUVEC migration when HUVECs were treated with conditioned medium from *LDLRAD2*-knockdown HL-60 cell supernatant at 0 and 24 h (*n* = 3). (B) *LDLRAD2*-overexpressing OCI-AML3 cell supernatant significantly increased HUVEC migration at 0 and 24 h (*n* = 3). (C and D) In the Matrigel tube formation assay, *LDLRAD2* knockdown supernatant reduced capillary-like tube formation, whereas *LDLRAD2* overexpression supernatant increased tube lengths. These effects were reversed by the glycolysis inhibitor 2-DG (*n* = 3). (E and F) Relative wound healing rates in HL-60 cells and OCI-AML3 cells. (G and H) Length of neovascularization in HL-60 cells and OCI-AML3 cells. Data are presented as mean ± SD from three independent biological experiments using independent conditioned-medium preparations. E–H were analyzed by one-way ANOVA followed by Tukey’s multiple-comparison test. ∗*p* < 0.05, ∗∗*p* < 0.01, and ∗∗∗*p* < 0.001 versus control; ^###^*p* < 0.001 versus the LDLRAD2 OE group. OE, overexpression; NC, negative control.

To investigate whether this glycolytic reprogramming contributes to the proangiogenic phenotype, we assessed the motility of HUVECs using a scratch-wound healing assay. We observed that the *LDLRAD2* knockdown supernatant from HL-60 cells inhibited wound healing (Figures 4A and 4E), whereas the supernatant containing overexpressed *LDLRAD2* from OCI-AML3 cells enhanced this process (Figures 4B and 4F). Given the capacity of endothelial progenitor cells to differentiate and assemble into vascular networks,^32^^,^^33^ we further investigated the role of LDLRAD2 in angiogenesis using a Matrigel-based tube formation assay. The results demonstrated that HUVECs cultured with conditioned medium from *LDLRAD2*-knockdown HL-60 supernatant exhibited minimal tubular structures (Figures 4C and 4G), whereas those exposed to *LDLRAD2*-overexpressing OCI-AML3 cell supernatant formed extensive capillary-like networks (Figures 4D and 4H). To assess whether the enhanced glycolysis mediated by LDLRAD2 is required for its proangiogenic effects, we treated HUVECs with 2-DG in the presence of *LDLRAD2*-overexpressing OCI-AML3 supernatant. This intervention abolished both cell migration and tube formation (Figures 4D and 4H), suggesting the dependence of LDLRAD2-driven angiogenesis on glycolysis.

### LDLRAD2 orchestrates AML infiltration through MTDH/PI3K/AKT axis-driven glycolytic enhancement

**Figure 5:**
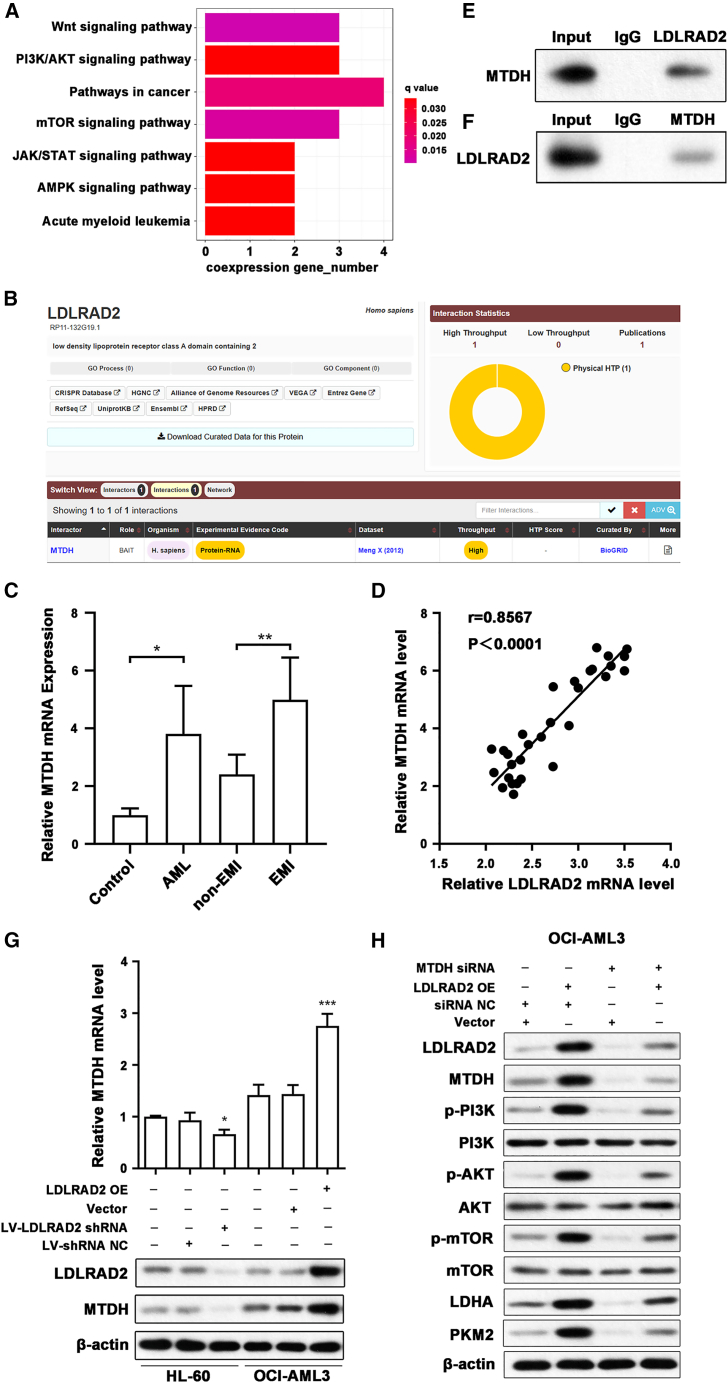
LDLRAD2 modulates the MTDH/PI3K/AKT axis to increase glycolysis and angiogenesis in AML (A) KEGG enrichment analysis of *LDLRAD2* coexpressed genes revealed significant enrichment in the Wnt and PI3K/AKT/mTOR signaling pathways. (B) The protein-protein interaction between LDLRAD2 and MTDH was predicted using the BioGRID database. The interaction network includes high throughput, low throughput, and publications. The pie chart shows the distribution of interaction evidence. (C) Compared with that in normal tissues (*n* = 10), *MTDH* mRNA expression was higher in AML samples obtained at EMI (*n* = 20) than in matched AML samples obtained at non-EMI (*n* = 20). (D) The *LDLRAD2* and *MTDH* mRNA expression levels were positively correlated in AML patient samples. (E) CoIP analysis confirmed the interaction between LDLRAD2 and MTDH. IgG was used as a negative control. (F) Reciprocal CoIP validating the interaction. (G) qPCR (top) and representative western blot analysis (bottom) demonstrated that LDLRAD2 knockdown downregulated MTDH expression, whereas LDLRAD2 overexpression upregulated MTDH expression. β-actin was used as a loading control. (H) Representative western blots showing that *LDLRAD2* overexpression increased the phosphorylation of PI3K, AKT, and mTOR, as well as glycolysis-related proteins (LDHA and PKM2), effects that were reversed by *MTDH* silencing. Nontransfected cells were used as a control, and β-actin was used as the loading control. Data are presented as mean ± SD unless otherwise indicated. C displays prespecified pairwise comparisons. Specifically, control versus AML and non-EMI versus EMI were compared using the Mann-Whitney U test. D was analyzed using Spearman’s rank correlation analysis. The quantitative qPCR data in G were analyzed by one-way ANOVA followed by Tukey’s multiple-comparison test. E, F, and H show representative immunoblots. ∗*p* < 0.05, ∗∗*p* < 0.01, and ∗∗∗*p* < 0.001 versus control; ^###^*p* < 0.001 versus the LDLRAD2 OE group. β-actin was used as the loading control. OE, overexpression; NC, negative control.

To elucidate the molecular mechanisms by which LDLRAD2 contributes to AML progression, we performed KEGG enrichment analysis of genes coexpressed with *LDLRAD2* (Pearson |r| > 0.3, *p* < 0.01) in the TCGA-AML cohort (Figure 5A). Notably, the Wnt and PI3K/AKT signaling pathways exhibited the most significant enrichment. The PI3K/AKT signaling pathway is known to increase glycolysis in tumors via various mechanisms, thereby influencing invasion, metastasis, and therapeutic resistance.^34^ Given the central role of these pathways in AML pathogenesis, we hypothesized that LDLRAD2 acts by interacting with key regulatory proteins.

Using the protein interaction database BioGRID, we identified metadherin (MTDH) as a protein interacting with LDLRAD2 (Figure 5B). To validate the clinical significance of this interaction, we analyzed *MTDH* mRNA expression levels in 40 AML patients and 10 healthy controls. Our results revealed significantly elevated *MTDH* expression in AML patients compared to controls (*p* < 0.05), with particularly high expression observed in EMI-AML subtypes relative to non-EMI-AML cases (*p* < 0.01; Figure 5C). Importantly, Spearman’s correlation analysis demonstrated a significant positive correlation between *LDLRAD2* and *MTDH* mRNA expression levels in AML patients (r = 0.8567, *p* < 0.001; Figure 5D), suggesting potential co-regulation or functional cooperation. This bioinformatic prediction was subsequently confirmed by CoIP experiments performed in OCI-AML3 cells, which provided direct evidence of physical interaction between LDLRAD2 and MTDH proteins (Figure 5E). A reciprocal CoIP assay using an anti-MTDH antibody further validated this specific interaction (Figure 5F).

*MTDH* is a well-characterized oncoprotein that modulates several key signaling cascades, such as the PI3K/AKT, NF-κB, and MAPK pathways, which collectively contribute to tumor development and metastasis.^35^^,^^36^^,^^37^^,^^38^ To assess this interaction, we designed three siRNAs targeting *MTDH* and a control siRNA. The most effective siRNA was chosen after validating the knockdown efficiency via qPCR and western blotting (Figure S1I–S1K). Intriguingly, both qPCR and Western blot analyses revealed that *LDLRAD2* knockdown reduced MTDH levels, whereas *LDLRAD2* overexpression increased MTDH levels (Figure 5G; Figure S3E). This reciprocal regulation suggests a potential feedback loop or mutual stabilization between the two proteins in cancer signaling. Additionally, *LDLRAD2* overexpression increased p-PI3K, *p*-AKT, and *p*-mTOR levels and the expression of glycolytic enzymes LDHA and PKM2; conversely, si-*MTDH* reversed these effects (Figure 5H; Figure S3F). Taken together, these results indicate that LDLRAD2 facilitates AML progression by enhancing glycolytic metabolism through the MTDH-mediated PI3K/AKT/mTOR signaling axis.

### LDLRAD2 drives EMI *in vivo* via glycolysis-enhanced angiogenesis through MTDH/PI3K/AKT pathway activation

**Figure 6:**
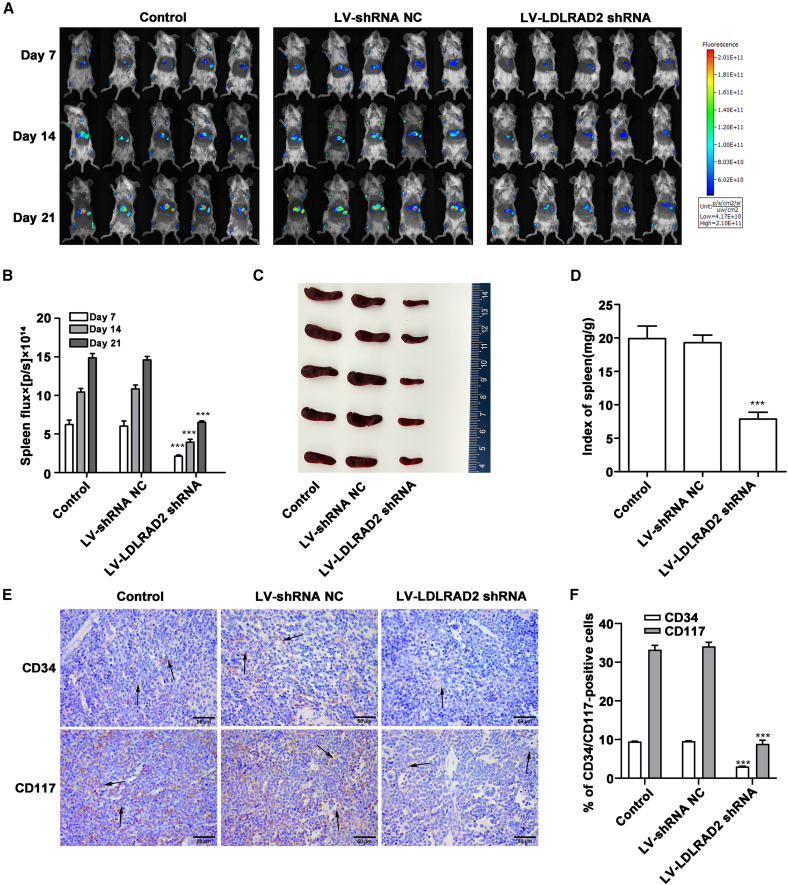
LDLRAD2 drives EMI *in vivo* by activating the MTDH/PI3K/AKT pathway to promote glycolysis and angiogenesis (Part 1) (A) Bioluminescence imaging over 21 days revealed a pronounced reduction in leukemic infiltration in the mice injected with LV-sh*LDLRAD2*-treated HL-60 cells compared with the control mice (*n* = 5). (B) Quantification of bioluminescence flux from serial *in vivo* imaging at days 7, 14, and 21 confirmed a decreased tumor burden in the LV-sh*LDLRAD2* group. (C) Macroscopic examination of spleen morphology revealed reduced leukemic infiltration upon *LDLRAD2* knockdown (*n* = 5). (D) The spleen index (normalized spleen-to-body weight ratio) was significantly lower in mice with *LDLRAD2*-knockdown cells. (E) Representative images of IHC staining for CD34 and CD117 expression at an original magnification of 400× (*n* = 3). (F) Quantification of the percentage of CD34-positive and CD117-positive cells measured from IHC staining (*n* = 3). Data are presented as mean ± SD. B shows quantitative bioluminescence measurements derived from serial *in vivo* imaging at days 7, 14, and 21, and each time point was analyzed separately by one-way ANOVA followed by Tukey’s multiple-comparison test. D was analyzed by one-way ANOVA followed by Tukey’s multiple-comparison test using five biologically independent mice per group. F was analyzed by one-way ANOVA followed by Tukey’s multiple-comparison test using three biologically independent mice per group; CD34 and CD117 were analyzed separately. ∗∗*p* < 0.01 and ∗∗∗*p* < 0.001 versus control. Scale bars, 50 μm. Arrows indicate positive cells. NC, negative control (continued in Figure 7).

To assess the functional effect of LDLRAD2 on AML progression *in vivo*, we conducted xenograft experiments using bioluminescence imaging to track HL-60 cell infiltration. During the 21-day observation period, mice injected with LV-sh*LDLRAD2*-transduced HL-60 cells exhibited a pronounced reduction in leukemic infiltration (Figure 6A), whereas mice injected with HL-60 cells exhibited prominent leukemic infiltration in the spleen. After the experiments were completed, all the groups of mice were euthanized. The tumor burden was further quantified via splenic bioluminescent flux (Figure 6B) and macroscopic spleen morphology assessments (Figure 6C), both of which revealed significantly attenuated leukemic infiltration in the LV-sh*LDLRAD2* group. Consistent with these findings, the spleen index was markedly lower in mice with *LDLRAD2* knockdown (Figure 6D), thus supporting a correlation between downregulated *LDLRAD2* expression, both impaired oncogenic potential and suppressed EMI.

**Figure 7:**
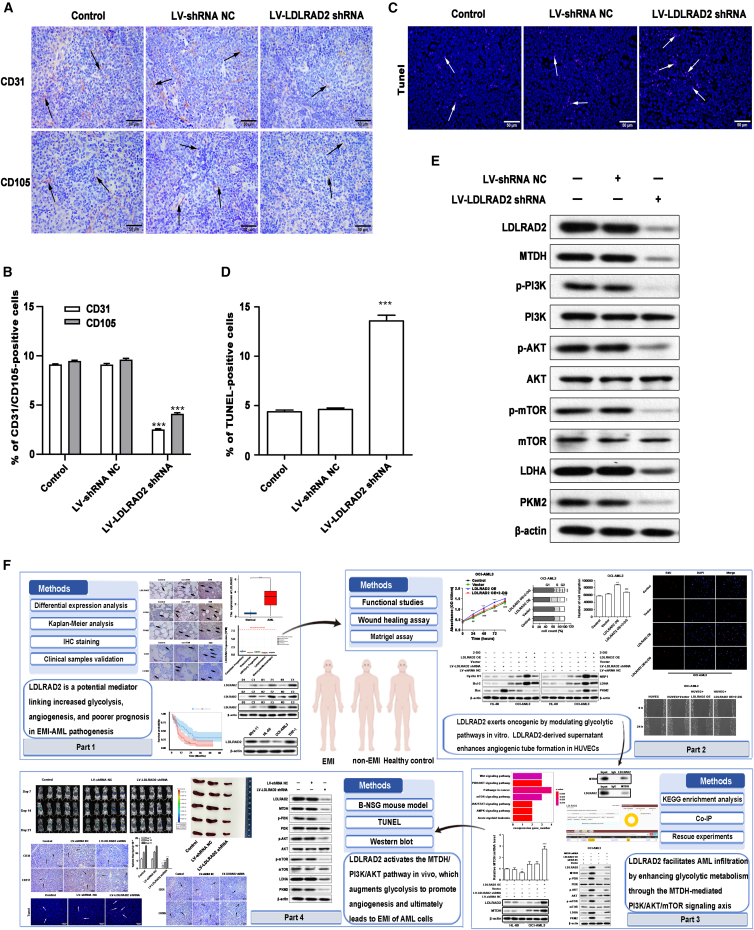
LDLRAD2 drives EMI *in vivo* by activating the MTDH/PI3K/AKT pathway to promote glycolysis and angiogenesis (Part 2) (A) Representative images of IHC staining for CD31 and CD105 expression at an original magnification of 400×. Scale bars, 50 μm. The arrows indicate positive cells. (B) Quantification of the percentage of CD31-positive and CD105-positive cells measured from IHC staining. (C) TUNEL staining revealed a significant increase in the number of apoptotic cells in the spleens of the mice injected with LV-sh*LDLRAD2*-transfected HL-60 cells compared with that observed in the control mice; original magnification of 400×. Scale bars, 50 μm. The arrows indicate tumor cells. (D) Percentage of TUNEL-positive cells. (E) Western blot analysis revealed downregulated LDLRAD2, MTDH, p-PI3K, *p*-AKT, *p*-mTOR, LDHA, and PKM2 expression in the spleens of *LDLRAD2*-knockdown mice, thereby indicating the suppression of the MTDH/PI3K/AKT signaling pathway and glycolysis. (F) The schematic illustrates the mechanisms of LDLRAD2 in EMI of AML via MTDH/PI3K/AKT signaling. Protein expression was measured via western blotting, and β-actin was used as a loading control. Data are presented as mean ± SD. B and D were analyzed by one-way ANOVA followed by Tukey’s multiple-comparison test using three biologically independent mice per group; in B, CD31 and CD105 were analyzed separately. E shows representative western blots of spleen lysates. ∗∗∗*p* < 0.001 versus control. Scale bars, 50 μm. Arrows indicate positive cells or TUNEL-positive cells. NC, negative control (continued from Figure 6).

IHC analysis was performed to detect the expression of hematopoietic progenitor markers CD34 and CD117 in spleen tissues. The results revealed that *LDLRAD2* knockdown significantly reduced the percentage of infiltrating CD34^+^ and CD117^+^ leukemic cells compared with the control treatment (Figures 6E and 6F), indicating impaired infiltration of AML cells into the spleen. To assess angiogenesis within the tumor microenvironment, we analyzed the expression of endothelial markers CD31 and CD105 in spleen tissues. Our analysis revealed that the percentage of CD31^+^ and CD105^+^ endothelial cells markedly decreased in the knockdown group, indicating the suppression of leukemia-associated angiogenesis (Figures 7A and 7B). Furthermore, TUNEL assays revealed a marked increase in the number of apoptotic cells within the spleens of mice injected with LV-sh*LDLRAD2* HL-60 cells compared to control mice (Figures 7C and 7D). To further evaluate the molecular effects of *LDLRAD2* knockdown, we analyzed the expression of proteins via western blotting. The results indicated that the expression of LDLRAD2, MTDH, p-PI3K, *p*-AKT, *p*-mTOR, LDHA, and PKM2 was downregulated in the spleen of *LDLRAD2*-knockdown mice (Figure 7E; Figure S3G). These findings demonstrated that *LDLRAD2* activates the MTDH/PI3K/AKT pathway *in vivo*, which augments glycolysis to promote angiogenesis and ultimately leads to EMI of AML cells (Figure 7F).

## Discussion

In this study, we identified LDLRAD2 as a critical regulator of EMI-AML, where its overexpression correlates with poor prognosis and fuels disease progression by altering the bone marrow microenvironment and angiogenesis. IHC analysis of EMI-AML patients highlighted the co-enrichment of LDLRAD2, glycolytic markers, and MVD, suggesting a coordinated role in disease progression. Mechanistically, LDLRAD2 activates the MTDH/PI3K/AKT/mTOR axis, promoting glycolysis and leading to angiogenesis (hallmarks of aggressive AML). Notably, AKT-driven glycolysis has been linked to chemoresistance in AML stem cells.^39^ However, upstream regulatory mechanisms remain incompletely understood. By identifying the LDLRAD2-MTDH interaction as a critical activator of this pathway, this not only explains the observed metabolic and angiogenic features in EMI-AML, but also highlights LDLRAD2 as a potential therapeutic target to disrupt AML progression at both the metabolic and microenvironmental levels.

*LDLRAD2*, a LDLR family member, has recently emerged as a promising gene implicated in tumor development and plays a crucial role in cellular signaling and adhesion.^40^ Dysregulated expression of LDLRAD2 has been observed in various cancers, wherein it promotes colorectal cancer (CC) and GC progression by modulating pathways such as Wnt/β-catenin and PI3K/AKT pathways.^24^^,^^25^^,^^41^ Nevertheless, the precise biological functions and molecular mechanisms of LDLRAD2 in AML (particularly EMI) remain to be elucidated. Furthermore, the interplay between LDLRAD2 and the tumor microenvironment requires further investigation to identify its broader implications in leukemogenesis. Our study confirms elevated LDLRAD2 expression in EMI-AML patients via clinical sample analysis, which drives AML progression by increasing cell proliferation, migration, and resistance to apoptosis, thus underscoring its oncogenic role. Notably, *in vivo* experiments further revealed that *LDLRAD2* knockdown attenuates splenic infiltration and EMI, thereby suggesting its specific involvement in aggressive disease dissemination. Collectively, our findings highlight LDLRAD2 as a critical regulator of AML pathogenesis (particularly in EMI) via its dual role in oncogenic potential and leukemic infiltration. This research not only advances the current understanding of LDLRAD2 in hematological malignancies, but also offers potential biomarkers for AML prognosis and the development of targeted therapeutic approaches.

A sufficient nutrient supply and a robust vascular network are prerequisites for AML infiltration.^42^^,^^43^ Angiogenesis is a critical process in the infiltration of hematological malignancies, including AML and acute lymphoblastic leukemia (ALL), thereby facilitating proliferation and migration.^44^^,^^45^^,^^46^ Antiangiogenic therapies have demonstrated limited efficacy, suggesting alternative mechanisms for vascular niche formation and infiltration.^47^^,^^48^^,^^49^ Similar to other aggressive malignancies, AML exhibits significant metabolic reprogramming features, with a hallmark feature involving the upregulation of aerobic glycolysis.^50^ Dysregulated glycolytic metabolism has been consistently linked to metastatic potential, disease relapse, and poor clinical outcomes.^51^ Moreover, lactate accumulation in the tumor microenvironment further drives the formation of new blood vessels that support cancer progression.^52^ Consistent with these findings, we revealed that the glycolytic pathway was activated in AML cells, thereby leading to increased lactate production and endothelial cell activation, which subsequently promoted vascular remodeling. Importantly, treatment with the glycolysis inhibitor, 2-DG, significantly suppressed AML-induced endothelial tube formation and reduced EMI *in vivo*. These findings suggest that glycolytic metabolites (rather than classical angiogenic factors) may play key roles in AML-mediated vascular niche formation and extramedullary spread. We acknowledge that our evidence for glycolytic reprogramming, while consistent, is indirect. Future studies employing direct flux measurement techniques such as Seahorse ECAR/OCR assays and 13C-glucose tracing will provide more definitive validation. Additionally, 2-DG is a broad-spectrum inhibitor of glycolysis. Future studies using more selective inhibitors targeting specific glycolytic enzymes will help elucidate the precise metabolic nodes regulated by the LDLRAD2.

Elevated MTDH expression has been linked to adverse clinical outcomes in malignant tumors such as breast cancer (BC) and CC, as evidenced in previous studies.^53^^,^^54^ Additionally, MTDH-mediated oncogenic effects are closely associated with dysregulation of the PI3K-AKT signaling cascade.^55^ This pathway is pivotal in regulating hematopoietic differentiation, and its aberrant activation is frequently implicated in the pathogenesis of hematological malignancies including AML and ALL.^56^^,^^57^ Interestingly, the direct connections between LDLRAD2, MTDH, and the PI3K/AKT pathway in hematological malignancies represent a previously underexplored connection. In this study, we confirmed the targeted relationship between LDLRAD2 and MTDH, LDLRAD2 upregulates MTDH expression, the expression of the PI3K/AKT pathway and glycolytic enzymes LDHA and PKM2 were strongly suppressed following si-*MTDH* treatment, thus highlighting their significance in AML progression. Additionally, the lengths of the generated HUVEC tubes were shortened owing to glycolysis inhibition. The dual function of LDLRAD2 (encompassing metabolic reprogramming and angiogenesis) underscores its strong association with EMI.

In conclusion, our study identified LDLRAD2 as a pivotal molecule in the regulation of the synergistic process known as “metabolic reprogramming-angiogenesis” in AML infiltration. The LDLRAD2–MTDH interaction and the subsequent activation of the PI3K/AKT/mTOR pathway underscores the dual value of this molecule as a prognostic biomarker and therapeutic target. Mechanistic research has demonstrated that *LDLRAD2* knockdown or glycolysis inhibition can effectively block the progression of AML. These findings not only elucidate the molecular mechanisms underlying the reorganization of the leukemia microenvironment but also provide a theoretical basis and therapeutic rationale for the development of precision treatment strategies for EMI by simultaneously targeting both metabolic and angiogenic pathways. To build upon these findings, future research should incorporate more physiologically relevant models, including patient-derived xenograft (PDX) systems and bone marrow stromal cell coculture. Additionally, rescue experiments and competitive engraftment studies will be important to fully elucidate the specificity of LDLRAD2 function and its role in EMI pathogenesis.

### Limitations of the study

While this study established a role for LDLRAD2 in EMI-AML, certain limitations should be noted. The *in vivo* experiments used the HL-60 cell line, which lacks common AML-associated genetic alterations and may not fully represent clinical heterogeneity. Future validation using more representative AML cell lines would strengthen the translational relevance. Additionally, the experimental models employed, including cell line-derived xenografts (CDX) and *in vitro* systems lacking stromal components, only partially recapitulate the complex microenvironment of human EMI. The incorporation of more physiologically relevant models, such as PDX and stromal co-culture systems, will be crucial for further understanding the role of LDLRAD2 *in vivo*. Mechanistically, the absence of rescue experiments after *LDLRAD2* knockdown and the reliance on indirect measurements of metabolic flux limit some mechanistic conclusions. Glycolysis inhibition experiments lack a direct “control vector + 2-DG” group. Nevertheless, control experiments using conditioned medium confirmed that the vector itself did not affect angiogenesis and that 2-DG inhibition was specific to *LDLRAD2* overexpression. Furthermore, although the interaction between LDLRAD2 and MTDH was validated by reciprocal CoIP in overexpression models, it was not performed in *LDLRAD2*-knockdown cells because of the risk of false-negative results arising from low endogenous protein expression. This remains an important direction for future investigation. Future work should also directly quantify glycolytic activity and explore the role of LDLRAD2 in AML cell homing.

## Resource availability

### Lead contact

Direct requests for further information, resources, and reagents to the lead contact, Desheng Kong (100863@hrbmu.edu.cn).

### Materials availability

All the unique/stable reagents generated in this study are available from the lead contact, with a completed material transfer agreement. This study did not generate new unique reagents.

### Data and code availability

Public datasets analyzed in this study are available from NCBI GEO: GSE116616, GSE107011, GSE6891, and GSE9476. TCGA-AML, TARGET AML, and GEPIA2 resources used in this study are publicly accessible and listed in the Key Resources Table. Newly generated source data underlying the quantitative analyses and uncropped western blot images with molecular weight markers have been deposited in Zenodo: https://doi.org/10.5281/zenodo.19528287 and are available upon reasonable request from the lead contact. This study did not generate custom code. Any additional information required to reanalyze the data reported in this paper is available from the lead contact upon reasonable request.

## Acknowledgments

This work was supported by the following funding sources: the Heilongjiang Postdoctoral Research Grant (21042220139), the Emerging Tumor Supportive Therapy Research Initiative (cphcf-2022-161), the Fourth Affiliated Hospital of Harbin Medical University Innovative Talent Fund (HYDSYCXRC202107), and the Heilongjiang Provincial Health Commission Science and Technology Program (20240303040046). The authors gratefully acknowledge financial assistance provided by these institutions.

## Author contributions

K.J. designed the study and wrote the manuscript; Y.Z., Q.G., J.L., H.Z., and S.F. performed experiments and analyzed data; C.J. and S.L. supervised the project and provided resources; D.K. reviewed the manuscript and secured funding. All authors have read and approved the final submitted manuscript.

## Declaration of interests

The authors declare no competing interests.

## STAR★Methods

### Key resources table

**Table:** 

REAGENT or RESOURCE	SOURCE	IDENTIFIER
**Antibodies**		

Cyclin D1	Wanleibio	Cat# WL01435a, RRID: AB_3665069
Bcl-2	Wanleibio	Cat# WL01556, RRID: AB_2904235
Bax	Wanleibio	Cat# WL01637, RRID: AB_2904236
β-actin	Wanleibio	Cat# WL01372, RRID: AB_2847841
Goat Anti-Rabbit IgG-HRP	Wanleibio	Cat# WLA023, RRID: AB_2890057
LDLRAD2	Thermo Fisher	Cat# PA5-101654, RRID: AB_2851088
MTDH	Affinity	Cat# DF13437, RRID: AB_2846456
LDHA	Wanleibio	Cat# WL03271, RRID: AB_3697327
PKM2	Zenbio	Cat# R381318
CD105	Abclonal	Cat# A19008, RRID: AB_2862500
CD31	Zenbio	Cat# 347526, RRID: AB_3719476
CD34	Abclonal	Cat# A19015, RRID: AB_2862507
CD117	Wanleibio	Cat# WL00125, RRID: AB_3675689
p-PI3K	Zenbio	Cat# 341468, RRID: AB_3675929
*p*-AKT	Wanleibio	Cat# WLP001a, RRID: AB_3665475
*p*-mTOR	Wanleibio	Cat# WL03694, RRID: AB_3676359
NRP1	Zenbio	Cat# R380865
PI3K	Wanleibio	Cat# WL02240, RRID: AB_3662845
AKT	Wanleibio	Cat# WL0003b, RRID: AB_2833233
mTOR	Wanleibio	Cat# WL02477, RRID: AB_3675748

**Biological samples**		

AML patient samples	Department of Hematology of the Fourth Affiliated Hospital of Harbin Medical University	Ethics Committee approval: 2024-ethic review-25
BMMC	Department of Hematology of the Fourth Affiliated Hospital of Harbin Medical University	Ethics Committee approval: 2024-ethic review-25

**Chemicals, peptides, and recombinant proteins**		

IMDM Medium	Procell	Cat# PM150510
HUVEC-specific Culture Medium	Cellverse	Cat# iCell-h110-001b
FBS	Tianhangbio	Cat# 11011-8611
PBS	Sangon	Cat# B548117
Mitomycin C	Sigma	Cat# M0503
Transwell Chamber	Labselect	Cat# 14342
Matrigel	Corning	Cat# 356234
Hematoxylin	Solarbio	Cat# H8070
DAB	Maixinbio	Cat# DAB-1031
Triton X-100	Beyotime	Cat# ST795
DAPI	Aladdin	Cat# D106471
ECL Substrate	Wanleibio	Cat# WLA006
PVDF Membrane	Millipore	Cat# IPVH00010
2-DG	Macklin	Cat# D807272

**Critical commercial assays**		

CCK-8 Cell Proliferation Assay Kit	Wanleibio	Cat# WLA074
Cell Cycle Assay Kit	Wanleibio	Cat# WLA010
Annexin V-light650/PI Cell Apoptosis Assay Kit	Wanleibio	Cat# WLA002
*In Situ* Apoptosis Detection Kit	Roche	Cat# 12156792910
BCA Protein Assay Kit	Wanleibio	Cat# WLA004
SDS-PAGE Rapid Gel Preparation Kit	Wanleibio	Cat# WLA013
Glucose Uptake Colorimetric Assay Kit	Wanleibio	Cat# WLA134
Lactate Colorimetric Assay Kit	Jianchengbio	Cat#A019
EdU Imaging Assay Kit	Kaijibio	Cat#KGA9604

**Deposited data**		

GSE116616	NCBI GEO	NCBI GEO: GSE116616
GSE107011	NCBI GEO	NCBI GEO: GSE107011
GSE6891	NCBI GEO	NCBI GEO: GSE6891
GSE9476	NCBI GEO	NCBI GEO: GSE9476
Quantitative source data and uncropped western blot images	Zenodo	Zenodo: 10.5281/zenodo.19528287

**Experimental models: Cell lines**		

HL-60	Shanghai Cell Bank, Chinese Academy of Sciences	RRID: CVCL_0002
THP-1	Shanghai Cell Bank, Chinese Academy of Sciences	RRID: CVCL_0006
OCI-AML3	Shanghai Cell Bank, Chinese Academy of Sciences	RRID: CVCL_1844
MV4-11	Shanghai Cell Bank, Chinese Academy of Sciences	RRID: CVCL_0064
HUVEC	Icellbio	RRID: CVCL_2959

**Experimental models: organisms/strains**		

B-NSG Mice	Wanleibio	NOD-Prkdcscid Il2rgtm1/Bcgen

**Oligonucleotides**		

GAPDH Forward Primer	ShangHai GenePharma Co., Ltd	GAAGGTGAAGGTCGGAGTCA
GAPDH Reverse Primer	ShangHai GenePharma Co., Ltd	GAGGTCAATGAAGGGGTCAT
LDLRAD2 Forward Primer	Jiangsu Genecefe Biotechnology Co., Ltd	TTTCCGTCTGGGACCTTGTG
LDLRAD2 Reverse Primer	Jiangsu Genecefe Biotechnology Co., Ltd	TCTGGAGGTATGGGCATCTGT
MTDH Forward Primer	Jiangsu Genecefe Biotechnology Co., Ltd	GAGAAGCCCAAACCAAAT
MTDH Reverse Primer	Jiangsu Genecefe Biotechnology Co., Ltd	ATCAGTCAGCACCTTATCAC

**Recombinant DNA**		

LDLRAD2 shRNA lentiviral vector	Jiangsu Genecefe Biotechnology Co., Ltd	5′-ccgGAAGTACAGATGCCCATACCTttcaagagaAGGTATGGGC ATCTGTACTTCGttttt-3′
LDLRAD2 overexpression plasmid	General Biosystems Co., Ltd	ATGGAGGCTTGTTGTCTTCTGCAGTTGCCCCAAAGGTTGCTCTT GCTGGGGGCAGCCGCCCTGACTGCAACTGCTTTGGAGACAGC CGACCTGGCGGAACTGTGCGGGCAGACGTGGCAGGGGGACG GGCTGCTGCTGCGCTCGCACGCCGCATCGCGCAGGTTCTACT TCGTGGCTCCGGACACCGACTGCGGGCTCTGGGTGCAGGCG GCAGCCCCCGGCGACCGGATCCGCTTCCAGTTCCGCTTCTTC CTGGTCTACAGCCTGACCCCCGCGCCCCCGGCGCTCAACACC TCCTCCCCGGCCCCGGCCGACCCGTGCGCCCCCGGCTCCTA CCTGCAGTTCTACGAGGGCCCGCCGGGGGCGCCCCGGCCCC TGGGGTCCCCACTGTGCGGCCTGAACATCCCGGTGCCTGTGG CATCCTCCGGACCCTTTCTAGGCCTGCGCCTGGTCACGAGAGG CCGCCAGCCCCGCGTGGACTTCGTGGGCGAAGTCACCTCTTTC CGTCTGGGACCTTGTGGTGCCTACTTCCGCTGCCAGAATGGCA GGTGCATCCCCTCAAGCCTCGTGTGTGACCCCTGGGGCATGGA CAACTGTGGCGATGGCAGTGACCAGGGCTCCTGGTCACCAGCT GACTGCAGAGGTCCCTCTCCGGTGCCCAGCCAGACAGGAAGTA CAGATGCCCATACCTCCAGATCCCTGACTCCCTCCCCAGCTCTC GGGTCTGCAGGATCCCTCTGGATTGCAGCTGAGAGGAGTTCCC CAGCAGGCAGGGACCCCACGAGACAAGACGCAGCTTTGGAAG GCTCCACTGAGTGA
Negative Control LDLRAD2 shRNA lentiviral Vector	Jiangsu Genecefe Biotechnology Co., Ltd	5′-ccgTTCTCCGAACGTGTCACGTttcaagagaACGTGACACGTT CGGAGAAttttt-3′
Si-MTDH interference fragment	Jtsbio	siMTDH-1: GCCGUAAUCAACCCUAUAUTTAUAUAGGGUUGA UUACGGCTT siMTDH-2: GGAACCAAUUCCUGAUGAUTTAUCAUCAGGAAU UGGUUCCTT siMTDH-3: GCUCUUCCAACUGGGAAAUTTAUUUCCCAGUU GGAAGAGCTT
Negative control siRNA	Jtsbio	UUCUCCGAACGUGUCACGUTTACGUGACACGUUCGGAGAATT

**Software and algorithms**		

TCGA-AML	TCGA	https://portal.gdc.cancer.gov/
TARGET AML	TARGET	https://ocg.cancer.gov/programs/target
GEPIA2	Z Lab, PKU	http://gepia2.cancer-pku.cn/
BioGRID	BioGRID	https://thebiogrid.org
GraphPad Prism 10.0	GraphPad Software	https://www.graphpad.com/
ImageJ	NIH	https://ImageJ.nih.gov/ij/
R-4.3.2	R-Project	https://www.r-project.org/
Image-Pro Plus 6.0	Media Cybernetics	https://mediacy.com/image-pro/
NovoExpress-1.6.3	Acea Biosciences	https://www.agilent.com/

### Experimental model and study participant details

#### Animal model

All animal procedures were approved by the Ethics Committee for Laboratory Animal Management of the Fourth Affiliated Hospital of Harbin Medical University (No. 2024-DWSYLLCZ-(24) and were performed in accordance with institutional guidelines and the ARRIVE recommendations. Six-week-old female NOD-Prkdcscid Il2rgtm1/Bcgen (B-NSG) mice (*n* = 15 total), whose average body weight ranged from 18 to 22 g at the start of the experiments, were purchased from Wanleibio and confirmed to be specific pathogen-free (SPF). The mice were group-housed (5 per cage) in individually ventilated cages under controlled conditions: 12 h light/12 h dark cycle, 22 ± 1°C temperature, and 45–55% humidity, with *ad libitum* access to sterilized food and water. The animals were acclimatized for 1 week before any procedures were performed. For the metastasis model, they were intravenously injected via the tail vein with LV-shRNA NC HL-60 cells, LV-shLDLRAD2 HL-60 cells (5×10^6^ cells/mouse) or control HL-60 cells suspended in 100 μL of sterile PBS. The sample size (*n* = 5 per group) was chosen on the basis of common practices in preliminary xenograft studies to observe a clear phenotypic effect, and was not predetermined by a formal power calculation. Humane endpoints were strictly defined and monitored daily: weight loss >20%, severe lethargy, inability to access food or water, signs of distress (hunching, piloerection), or the presence of a large palpable tumor burden. No animals met humane endpoints before the planned experimental endpoint.

#### Cell culture

The human AML cell lines THP-1 (RRID: CVCL_0006), MV4-11 (RRID: CVCL_0064), OCI-AML3 (RRID: CVCL_1844), and HL-60 (RRID: CVCL_0002) were obtained from the Shanghai Cell Bank, Chinese Academy of Sciences (Shanghai, China). All cell lines were confirmed to be free of mycoplasma contamination, and their genetic identities were verified by short tandem repeat (STR) profiling. To maintain experimental consistency, cells were passaged for no more than 1 month or 10 passages before a new aliquot was thawed. HL-60 cells were cultured in Iscove’s modified Dulbecco’s medium (IMDM, Procell, Wuhan, China) supplemented with 20% fetal bovine serum (FBS, Tianhangbio, Zhejiang, China) at 37°C in a humidified incubator containing 5% CO2. OCI-AML3 cells were maintained in IMDM containing 15% FBS under the same conditions. Human umbilical vein endothelial cells (HUVECs) (RRID: CVCL_(2959) were acquired from Icellbio. HUVECs were cultivated in specific culture medium in an incubator at 37°C with 5% CO_2_. According to RRID-linked cell line records, the sex of the cell lines used in this study was as follows: THP-1, male; MV4-11, male; OCI-AML3, male; HL-60, female; and HUVECs, female. Sex information was recorded for the experimental models used in this study; however, the study was not designed or powered to evaluate sex-based differences, and no sex-stratified analyses were performed.

### Method details

#### Bioinformatics analysis

Publicly available transcriptomic and clinical datasets were retrieved from GEO, TCGA, TARGET, and GEPIA2, as listed in the Key Resources Table. The GSE116616 dataset served as the training set. Cross-database validation of *LDLRAD2* expression in AML was performed using GEPIA2, which integrates TCGA-AML and GTEx data. Independent validation was further performed using a TARGET AML subset together with the GSE9476 dataset. The GSE107011 dataset was utilized to analyze *LDLRAD2* expression across 29 normal hematopoietic compartments. The GSE6891 dataset, which includes 537 AML patients with *FLT3*, *IDH1*, and *NPM1* mutation annotations, was analyzed to assess expression correlation with genetic subtypes. Overall survival (OS) and leukemia-free survival (LFS) were analyzed in the in-house AML cohort using Kaplan‒Meier curves, and the prognostic value of *LDLRAD2* was further assessed in independent public cohorts. KEGG enrichment analysis of *LDLRAD2*-coexpressed genes in the TCGA-AML cohort was performed in R (version 4.3.(2) using genes with Pearson |r| > 0.3 and *p* < 0.01. Candidate protein–protein interactions were explored using BioGRID.

#### Patient cohort and sample acquisition

Peripheral blood and bone marrow samples were obtained from 40 patients diagnosed with AML at the Department of Hematology, the Fourth Affiliated Hospital of Harbin Medical University between July 2024 and July 2025. For the EMI comparison, patients were classified as EMI-AML (*n* = 20) or non-EMI-AML (*n* = 20). For analyses of *LDLRAD2* expression across disease course, samples were also grouped according to the time of collection as newly diagnosed (*n* = 15), complete remission or complete remission with incomplete hematologic recovery (CR/CRi; *n* = 10), or relapsed disease (*n* = 15). Bone marrow samples from 10 healthy volunteers without hematologic disease collected during the same period were used as controls. The detailed demographic and clinical characteristics of AML patients and healthy donors are summarized in Table S1 and Table S2, respectively. Ethnicity data were not collected in this study. This study was approved by the Ethics Committee of the Fourth Affiliated Hospital of Harbin Medical University (No. 2024-ethic review-25), and written informed consent was obtained from all participants in accordance with the Declaration of Helsinki. The inclusion criteria for AML patients included patients who were newly diagnosed at ≥18 years of age and whose diagnosis was confirmed by bone marrow morphology (blast count ≥20%), immunophenotyping, cytogenetics, and molecular biology according to the 2016 WHO classification of myeloid neoplasms and acute leukemia. The exclusion criteria included concurrent active malignancies, severe hepatic/renal dysfunction (ALT/AST >3×ULN, Cr > 2×ULN), coagulopathy, pregnancy, or inability to provide informed consent or follow-up. Diagnostic classification was performed according to both the French-American-British (FAB) and World Health Organization (WHO) classification systems for hematopoietic malignancies.^58^^,^^59^

#### Plasmid construction and lentivirus infection

To validate the knockdown specificity, three independent shRNAs targeting different regions of human *LDLRAD2* mRNA (sh*LDLRAD2*-1, sh*LDLRAD2*-2, and sh*LDLRAD2*-(3) and a shNC were designed and synthesized by Jtsbio (Wuhan, China). The lentiviral overexpression plasmid containing the full-length human *LDLRAD2* coding sequence and the *LDLRAD2*-targeting shRNA lentiviral vector were synthesized by Jiangsu Genecefe Biotechnology Co., Ltd (Jiangsu, China) and General Biosystems Co., Ltd (Anhui, China). A scrambled shRNA lentiviral vector and an empty plasmid were used as negative controls. Additionally, siRNA-mediated knockdown was performed using si*MTDH* (a specific siRNA targeting human *MTDH*) and a negative control siRNA, both of which were purchased from Jtsbio (Wuhan, China). The detailed sequence information for all of the vectors, plasmids and siRNAs is listed in KEY RESOURCES TABLE. For overexpression, OCI-AML3 cells in optimal growth conditions were transfected with the *LDLRAD2* plasmid. For knockdown, HL-60 cells were infected with *LDLRAD2* shRNA lentiviral particles according to the manufacturers’ instructions. For siRNA transfection, OCI-AML3 cells were transfected with si-*MTDH* or negative control siRNA according to the manufacturer’s instructions. After 48 h, the cells were collected for downstream analysis. The efficiency of *LDLRAD2* overexpression or knockdown was confirmed via qPCR and Western blotting.

#### Total RNA extraction and quantitative real-time PCR

The expression levels of *LDLRAD2* and *MTDH* transcripts were determined using qRT-PCR. The primer sequences are listed in KEY RESOURCES TABLE.

#### Cell proliferation analysis

Cell viability was initially assessed using the Cell Counting Kit-8 assay (CCK-8, Wanleibio, China). Briefly, transfected HL-60 and OCI-AML3 cells (5 × 10^3^ cells/well) were seeded in a 96-well plate with 200 μL of culture medium and incubated at 37 °C with 5% CO_2_ for 0, 24, 48, 72 or 96 h. After incubation, 10 μL of CCK-8 solution was added to each well and incubated in the dark for 2 h. The optical density (OD) was measured at 450 nm using a microplate reader (800 TS, USA). Cell viability was calculated as follows: cell viability rate (%) = (AEX - AOE)/(ANC - AOE) × 100%. Moreover, the cell inhibition rate was calculated as follows: cell inhibition rate (%) = 1 - cell viability rate (%). To directly measure cell proliferation independent of metabolic activity, 5-ethynyl-2′-deoxyuridine (EdU) incorporation assays were performed using an EdU Imaging Assay Kit (Kaijibio, Guangzhou, China) according to the manufacturer’s instructions. Briefly, the cells were incubated with 50 μM EdU for 2 h, fixed, permeabilized, and stained. The percentage of EdU-positive cells was quantified using fluorescence microscopy.

#### Flow cytometric analysis of the cell cycle and apoptosis

Cell cycle distribution and apoptosis were assessed using a flow cytometer (NovoCyte, USA). For cell cycle analysis, the cells were stained with propidium iodide (PI). A sequential gating strategy was applied: First, the main cell population was gated on FSC-H vs. SSC-H to exclude debris and large aggregates (P1). Cells within P1 were then plotted on PI-A vs. PI-H, and a narrow gate was set to select single nuclei on the basis of pulse processing. Finally, the DNA content histogram was generated from the PI-A signal of the single-nucleus population for cell cycle phase distribution analysis using NovoExpress software. Apoptosis was assessed using the Annexin V-light650/PI Cell Apoptosis Assay Kit (Wanleibio, China) following the manufacturer’s protocol.

#### Transwell migration assays

The migratory capacities of HL-60 and OCI-AML3 cells were assessed using a Transwell system. Briefly, transfected cells were resuspended in serum-free RPMI-1640 medium (1 × 10^5^ cells/mL) and seeded into the upper chamber (200 μL/well), whereas the lower chamber was filled with RPMI-1640 medium containing 10% FBS (800 μL/well). After 48 h, the migrated cells in the lower chamber were counted, and the nonmigrated cells were removed. The number of migration cells in each chamber was quantified for further analysis.

#### Western blotting analysis

Total protein was extracted from cells or tissues using RIPA buffer and quantified using a BCA Protein Assay Kit. Equal amounts of protein (40 μg per lane) were separated by SDS-PAGE using 5% stacking gels and 7%–12% resolving gels, and then transferred onto PVDF membranes. After blocking with 5% BSA, the membranes were incubated overnight at 4°C with the indicated primary antibodies listed in the Key Resources Table, followed by HRP-conjugated goat anti-rabbit secondary antibodies (1:5000). Protein bands were visualized using enhanced chemiluminescence. Representative western blot images are shown in the figures, and unedited, uncropped western blot images are provided in the supplemental data files.

#### Wound healing assay

HUVECs were cultured until they reached 95–100% confluence at 37 °C and treated with 1 mg/mL Mitomycin C for 1 h. A uniform scratch was made using a 200 μL pipette tip, and the suspended cells were removed by washing with PBS. HL-60 cells were infected with an *LDLRAD2*-silencing lentiviral vector, and OCI-AML3 cells were transfected with an *LDLRAD2*-overexpressing plasmid. After 48 h, the cell supernatants were collected as conditioned media. The collected supernatants (from HL-60 or OCI-AML3 cells) were added to the HUVECs and incubated for 24 h at 37 °C. Migration was assessed using imaging at 0 h and 24 h by using an optical microscope (Olympus Corporation) and quantified by using ImageJ.

#### *In vitro* matrigel HUVEC tube formation assays

The Matrigel-based tube formation assay was performed by coating a 96-well plate with 50 μL of thawed Matrigel per well and allowing it to solidify at 37 °C for 1 h. HUVECs (1 × 10^4^ cells/well) were seeded and treated with conditioned supernatant from either *LDLRAD2*-knockdown HL-60 cells or *LDLRAD2*-overexpressing OCI-AML3 cells. After 6 h of incubation at 37 °C, tube formation was assessed under an inverted microscope, and the vascular network length was quantified for analysis.

#### Coimmunoprecipitation (CoIP) analysis

OCI-AML3 cells were lysed to extract total protein, which was quantified by a BCA assay. For CoIP, protein extracts were incubated overnight at 4 °C with rotation, and either an anti-LDLRAD2 antibody (1:120, Thermo Fisher) or an anti-MTDH antibody (1:100, Affinity) was used for reciprocal validation. Protein A/G beads were then added and incubated for an additional 2 h. The precipitated immune complexes were extensively washed, eluted, and separated by SDS‒PAGE. Proteins were transferred to a PVDF membrane and probed with the indicated primary antibodies: anti-MTDH (1:500, Affinity) or anti-LDLRAD2 (1:200, Thermo Fisher). After the membranes were incubated with HRP-conjugated secondary antibodies (1:5000, Wanleibio), protein interactions were detected by enhanced chemiluminescence (ECL).

#### *In vivo* bioluminescence imaging of xenografts

*In vivo* bioluminescence imaging was performed on days 0, 7, 14, and 21 post injection to systematically track the migration and infiltration of tumor cells in the mice. For imaging, the mice were anesthetized by induction with 3–4% isoflurane in oxygen and maintained under 1.5–2% isoflurane using a precision vaporizer. The experimental unit was a single mouse. No specific strategies were employed to control for potential confounding factors such as cage location or measurement order. At the experimental endpoint, mice were euthanized by carbon dioxide asphyxiation followed by cervical dislocation, in accordance with the AVMA Guidelines for the Euthanasia of Animals. Spleens were harvested, weighed, and used for subsequent histological and protein analyses. The spleen index was calculated as spleen weight divided by body weight.

#### Immunohistochemistry (IHC) staining

The bone marrow biopsies from AML patients and mouse spleen tissues were fixed in 10% neutral buffered formalin, paraffin-embedded, and sectioned at 4 μm thickness for IHC analysis. After standard deparaffinization and antigen retrieval, tissue sections were incubated with primary antibodies against LDLRAD2 (Thermo Fisher, USA), LDHA (Wanleibio, China), PKM2 (Zenbio, China), CD105 (Abclonal, China), CD31 (Zenbio, China), CD34 (Abclonal, China), and CD117 (Wanleibio, China), followed by appropriate HRP-conjugated secondary antibody (Thermo Fisher, USA) and DAB (Maixinbio, China) chromogenic development, with hematoxylin (Solarbio, China) counterstaining. Quantitative analysis of IHC staining was performed by two independent investigators who were blinded to the sample groups, with discrepancies resolved by consensus or adjudication by a senior pathologist. For both human bone marrow and mouse spleen tissues, five representative high-power fields (HPF, 400×) per slide were evaluated under an OLYMPUS microscope (Tokyo, Japan). Specific quantification methods were tailored to the markers and tissue types. For human bone marrow samples, the expression of LDLRAD2, LDHA, and PKM2 was assessed by determining the percentage of positive cells (% positive cells), which was calculated as (number of positive cells/total number of cells) × 100% across five HPFs, with cytoplasmic DAB staining considered positive. To assess the MVD using CD31 and CD105 in human bone marrow: the percentage of CD31-positive or CD105-positive cells (as described above), which was measured using ImageJ software (NIH). For mouse spleen tissue samples, the expression of CD31, CD105, CD34, and CD117 was quantified by determining the percentage of positive cells (% positive cells) among the five HPFs. For the angiogenesis markers CD31 and CD105 in the mouse spleen, three areas with the highest vascular density were first identified at low magnification (40×), and positive vessels were then counted at 400× to calculate the percentage.

#### Glycolysis assay

To investigate the effect of *LDLRAD2* overexpression on glycolysis in OCI-AML3 cells, the cells were transfected with an *LDLRAD2* overexpression plasmid (General Biosystems, Anhui, China) following the manufacturer’s protocol. After 48 h of transfection, the cells were treated with 5 mM 2-deoxy-D-glucose (2-DG, Macklin, Shanghai, China) for 24 h to inhibit glycolysis. Glucose consumption and lactate production were subsequently measured using a Glucose Uptake Colorimetric Assay Kit (Wanleibio, China) and Lactate Colorimetric Assay Kit (Jianchengbio, China), respectively, following the manufacturers’ protocols. Supernatants were collected at designated time points, and glucose consumption was determined by quantifying extracellular glucose depletion, whereas lactate production was measured to reflect glycolytic levels. The data were normalized to the cell number or protein content to ensure comparability.

#### Terminal deoxynucleotidyl transferase-mediated dUTP nick end labeling (TUNEL) assay

Apoptosis in mouse spleen tissues was detected via TUNEL staining using an *In Situ* Cell Death Detection Kit (Roche, Switzerland) following the manufacturer’s guidelines. Tissue sections were processed through deparaffinization, permeabilization with 0.1% Triton X-100 (Beyotime, China), and TUNEL reaction incubation. Nuclei were counterstained with DAPI (Aladdin, China), and slides were observed using a microscope (Olympus, Tokyo, Japan) at a magnification of 400×. Apoptotic cells were assessed in three representative fields per sample.

#### Ethics approval and consent to participate

This study was performed in accordance with the Declaration of Helsinki and approved by the Ethics Committee of the Fourth Affiliated Hospital of Harbin Medical University (No. 2024-ethic review-25). Each patient signed an informed consent form before participating in this study. All animal experiments were approved by the Ethics Committee for Laboratory Animal Management of the Fourth Affiliated Hospital of Harbin Medical University (No. 2024-DWSYLLCZ-(24) and were performed in accordance with institutional guidelines for the care and use of laboratory animals.

### Quantification and statistical analysis

Statistical analyses were performed using GraphPad Prism 10.0 (GraphPad Software, San Diego, CA, USA). Data are presented as mean ± SD unless otherwise indicated. Normality and homogeneity of variance were assessed using the Shapiro–Wilk and Brown–Forsythe tests, respectively. For comparisons between two groups, an unpaired two-tailed Student’s *t* test was used when data met assumptions of normality and equal variance; otherwise, the Mann–Whitney U test was applied. For comparisons among three or more groups, one-way ANOVA followed by Tukey’s multiple-comparison test was used for normally distributed data, whereas the Kruskal–Wallis test followed by Dunn’s multiple-comparison test was used when parametric assumptions were not met. For experiments involving two independent variables, including time course proliferation assays, two-way ANOVA followed by Bonferroni posttests was used. Categorical variables in patient cohorts were analyzed using Fisher’s exact test or the chi-square test, as appropriate. Survival curves were generated using the Kaplan–Meier method and compared using the log rank test. Correlations between *LDLRAD2* and *MTDH* mRNA expression were assessed using Spearman’s rank correlation analysis. All tests were two-sided, and *p* < 0.05 was considered statistically significant. No animals or data points were excluded from analysis. Exact statistical tests, biological units, sample sizes, and additional statistical details are provided in the figure legends and Table S3.

## Supplemental information

Document S1. Figures S1–S3 and Tables S1–S2

Table S3. Statistical analysis summary for Figures 1–7 and Figures S1–S3
