# Supplementary material for: LDLRAD2 drives glycolysis and angiogenesis to promote extramedullary infiltration in acute myeloid leukemia
Source: iScience. 2026 May 20;29(6):115987. doi: 10.1016/j.isci.2026.115987 (PMC13214308; doi:10.1016/j.isci.2026.115987)
Supplement: Document S1. Figures S1–S3 and Tables S1–S2 [file mmc1.pdf]

iScience, Volume *29*

Supplemental information

LDLRAD2 drives glycolysis and angiogenesis to promote extramedullary infiltration in acute myeloid leukemia

Kexin Jin, Yang Zhao, Qiang Guo, Jingyi Lin, Hongli Zhao, Shengjin Fan, Chuiming Jia, Shuchuan Liu, and Desheng Kong

SUPPLEMENTAL INFORMATION

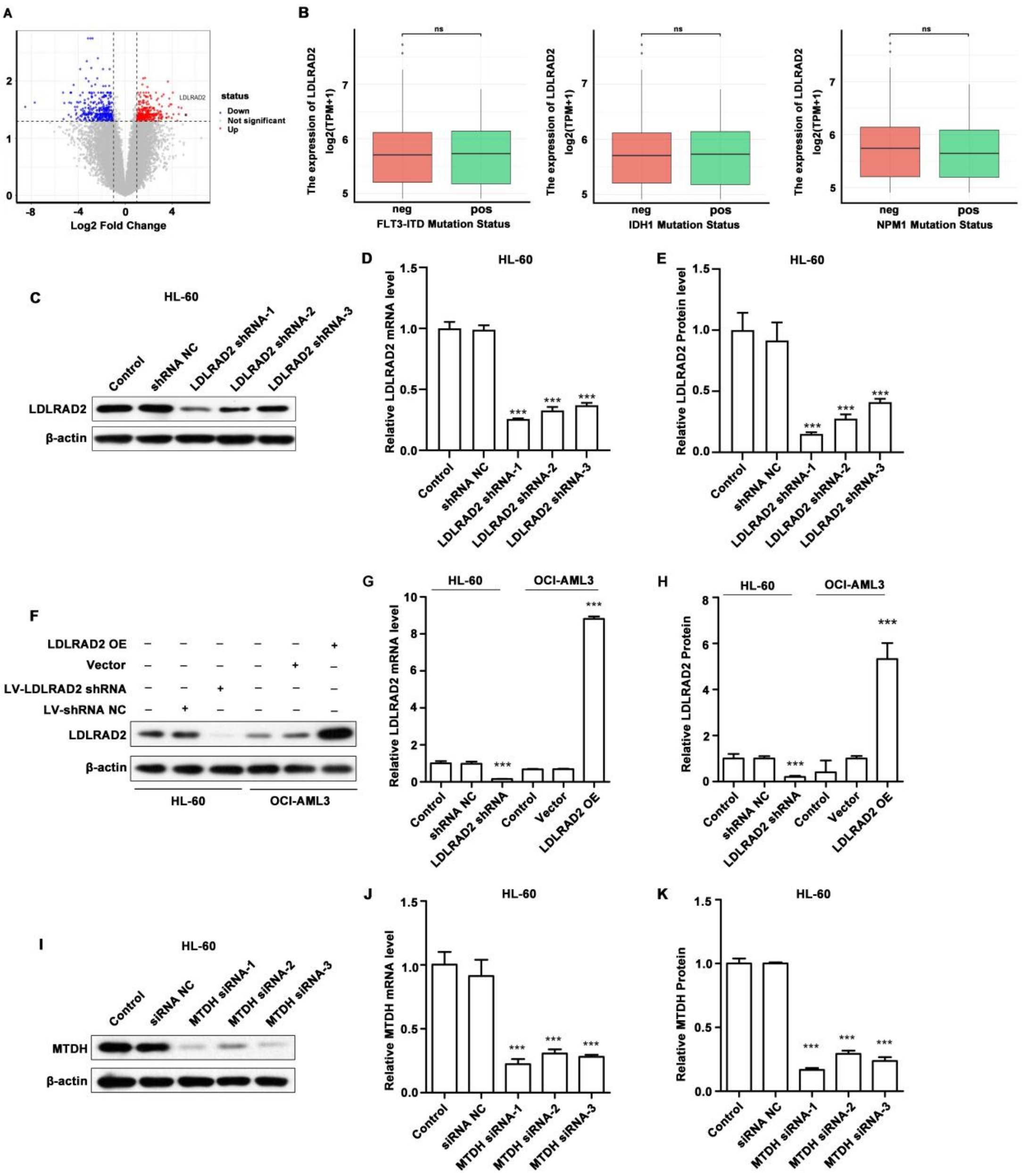

Figure S1. Regulation of *LDLRAD2* and *MTDH* expression in AML cell lines and their intermolecular interactions. Related to Figure 1, Figure 3, and Figure 5.

(A) *LDLRAD2* mRNA expression was significantly greater in AML samples (n = 8) than in healthy control samples (n = 4) (log2FC > 1, $P < 0.05$; GSE116616 dataset).

(B) The mRNA expression between *LDLRAD2* and *FLT3* (left), *IDH1* (middle), or *NPM1* (right) mutation.

(C) Upon transfection with three distinct shRNAs (*LDLRAD2* shRNA#1-3), a reduction in LDLRAD2 protein expression in AML cells compared with negative control cells was confirmed via Western blot analysis. β-actin was used as a loading control.

(D-E) Relative *LDLRAD2* mRNA (D) and protein (E) levels in HL-60 cells treated with shRNAs targeting *LDLRAD2* (shRNA-1, shRNA-2, or shRNA-3). qRT-PCR was used to validate the reduction in *LDLRAD2* mRNA levels after shRNA transfection. The most effective shRNA

(*LDLRAD2*-shRNA#1) was selected for subsequent experiments.
(F) Western blot analysis of LDLRAD2 protein levels in HL-60 and OCI-AML3 cells. β-actin was used as a loading control.
(G-H) Relative *LDLRAD2* mRNA (G) and protein (H) levels in HL-60 and OCI-AML3 cells treated with lentiviral shRNA-mediated knockdown and overexpression plasmids.
(I) Upon transfection with three distinct siRNAs (si-MTDH#1-3), a reduction in MTDH protein expression in AML cells compared with negative control cells was confirmed via Western blot analysis. β-actin was used as a loading control.
(J-K) Relative *MTDH* mRNA (J) and protein (K) levels in HL-60 cells treated with siRNAs targeting *MTDH* (siRNA-1, siRNA-2, or siRNA-3). qRT-PCR was used to validate the reduction in *MTDH* mRNA levels after siRNA transfection. The most effective siRNA (si-MTDH#1) was selected for subsequent experiments.
Nontransfected cells served as a control. Data are presented as mean ± SD. Panel A shows database-derived differential expression analysis from GSE116616 as reported by the dataset. Quantitative panels D, E, G, H, J, and K were analyzed by one-way ANOVA followed by Tukey's multiple-comparison test using three independent biological experiments, unless otherwise indicated. $^{**}P < 0.01$, $^{***}P < 0.001$ versus NC or control, as indicated. NC, negative control; OE, overexpression.

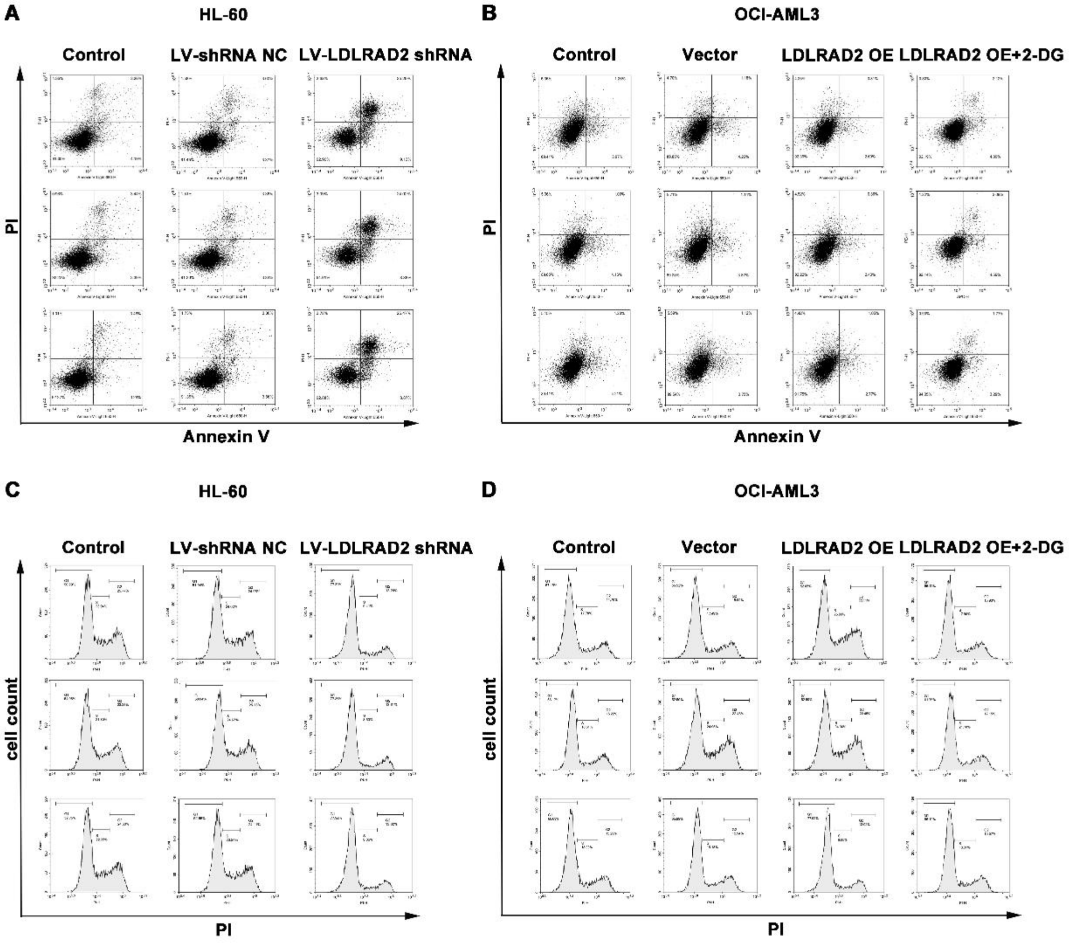

Figure S2. Flow cytometry analysis of apoptosis and cell cycle distribution in AML cells. Related to Figure 3.

(A-B) Representative flow cytometry plots of apoptosis in HL-60 (A) and OCI-AML3 (B) cells. Q2-4: early apoptotic cells; Q2-2: late apoptotic cells. The sum of the percentages of apoptotic cells in the Q2-4 and Q2-2 quadrants represents the percentage of total apoptotic cells (n = 3). (C-D) Representative flow cytometry plots of the cell cycle analysis of HL-60 (C) and OCI-AML3 (D) cells. (n = 3).

PI, propidium iodide; NC, negative control; OE, overexpression.

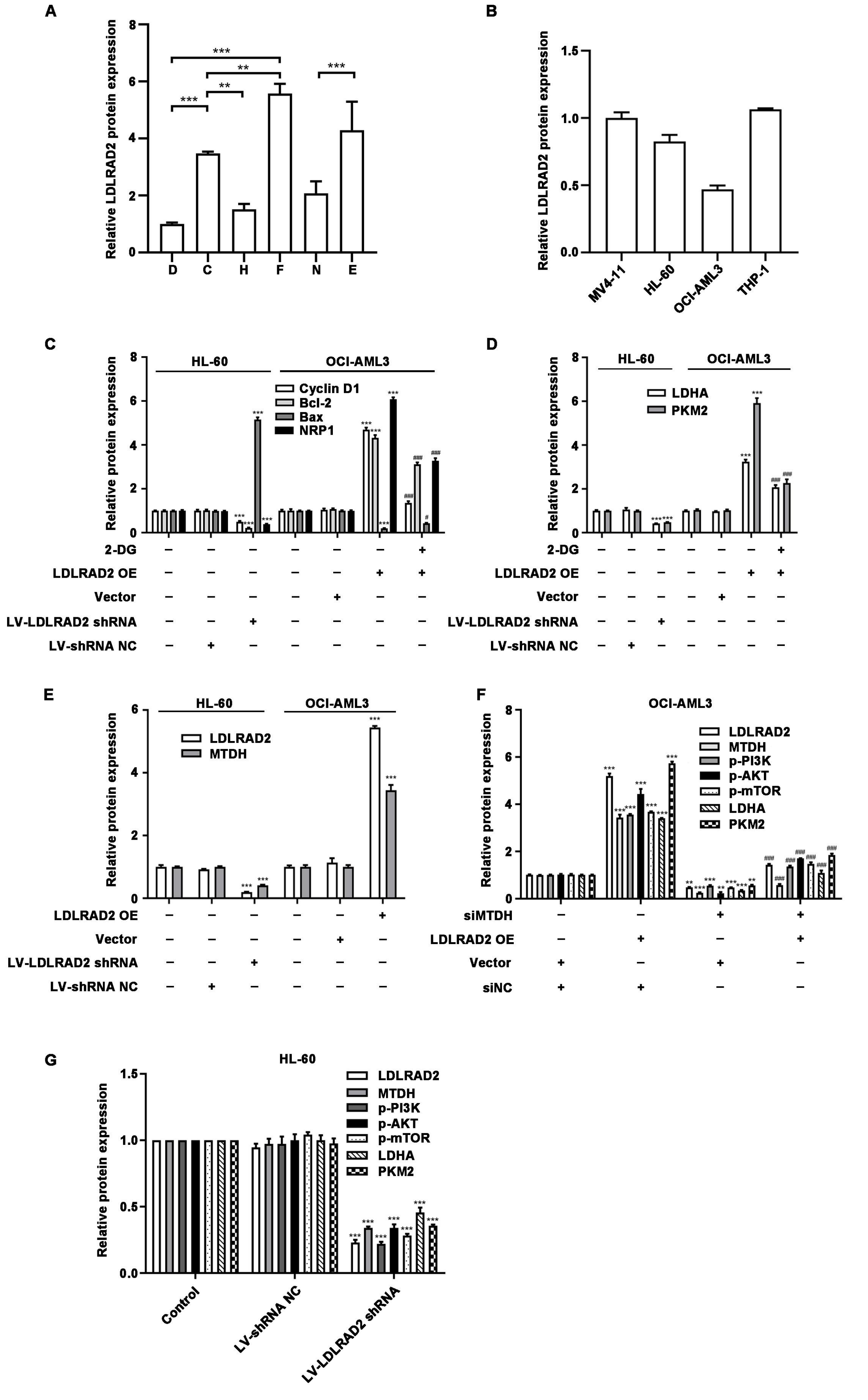

Figure S3. Effects of LDLRAD2 modulation on multiple protein. Related to Figure1,

Figure 4, Figure 5, and Figure 7.

(A) Relative protein expression levels of LDLRAD2 in clinical BMMC samples.

(B) Relative protein expression levels of LDLRAD2 in AML cell lines.

(C) Relative protein expression levels of Cyclin D1, Bcl-2, Bax, and NRP1 in HL-60 and OCI-AML3 cells. (n = 3).

(D) Relative protein expression levels of LDHA and PKM2 in HL-60 and OCI-AML3 cells. (n = 3)

(E) Relative protein expression levels of LDLRAD2 and MTDH in HL-60 and OCI-AML3 cells. (n = 3).

(F) Relative protein expression levels of LDLRAD2, MTDH, p-PI3K, p-AKT, p-mTOR, LDHA, and PKM2 in OCI-AML3 cells. (n = 3).

(G) Relative protein expression levels of LDLRAD2, MTDH, p-PI3K, p-AKT, p-mTOR, LDHA, and PKM2 in spleen tissues. (n = 3).

Nontransfected cells served as a control. Quantitative data are presented as mean ± SD. Panel A was analyzed using one-way ANOVA followed by Tukey's multiple-comparison test across the displayed clinical sample groups. Panels B–F were analyzed within each experimental background using one-way ANOVA followed by Tukey's multiple-comparison test with three independent biological experiments. Panel G was analyzed using one-way ANOVA followed by Tukey's multiple-comparison test with three biologically independent mice per group. $^{**}P < 0.01$ and $^{***}P < 0.001$ versus control; $^{\#}P < 0.05$ and $^{\#\#\#}P < 0.001$ versus the LDLRAD2 OE group, as indicated. D1: Control1, D2: Control2, D3: Control3, C1: Diagnosis1, C2: Diagnosis2, C3: Diagnosis3, H1: CR1, H2: CR2, H3: CR3, F1: Relapse1, F2: Relapse2, F3: Relapse3, N1: non-extramedullary infiltration1, N2: non-extramedullary infiltration2, N3: non-extramedullary infiltration3, E1: extramedullary infiltration1, E2: extramedullary infiltration2, E3: extramedullary infiltration3, NC: negative control, OE: overexpression.

Table S1. Comparison of clinical manifestations and laboratory features between AML patients with low and high *LDLRAD2* expression. Related to Figure 1.

**Patient's parameters**	***LDLRAD2* expression**		***P* Value**
	***LDLRAD2*^{Low} (n=20)**	***LDLRAD2*^{High} (n=20)**	
**Sex (male/female)**	10/10	11/9	0.7515
**Median age (range), years**	52(27-71)	55(39-78)	0.3859
**Median WBC (range), ×10^9/L**	26.88(0.97-180)	46.05(1.08-228.7)	0.3273
**Median hemoglobin (range), g/L**	85.85(56-121)	83.55(43-133)	0.7208
**Median platelets (range), ×10^9/L**	72.65(15-466)	64.30(1-203)	0.4986
**FAB classification**			0.6606
M1	2(5%)	4(10%)	
M2	5(13%)	6(15%)	
M4	4(10%)	5(13%)	
M5	5(13%)	4(10%)	
M6	4(10%)	1(3%)	
**Karyotype classification**			0.5107
Favorable	7(18%)	6(15%)	
Intermediate	7(18%)	10(25%)	
Poor	6(15%)	3(8%)	
No data	0(0%)	1(3%)	
**Karyotype**			0.4585
Normal	7(18%)	6(15%)	
t (8;21)	1(3%)	4(10%)	
inv (16)	2(5%)	3(8%)	
Complex	3(8%)	4(10%)	
Others	7(18%)	3(8%)	
**Gene mutation**			
FLT3-ITD (+/-)	3/15	7/11	0.1366
NPM1 (+/-)	5/13	4/15	0.7140
CEBPA (+/-)	5/14	3/16	0.6928
RUNX1 (+/-)	3/14	6/12	0.4430
IDH1/2 (+/-)	2/15	5/14	0.4080
DNMT3A (+/-)	1/13	4/14	0.3547

Abbreviations: AML, acute myeloid leukemia; WBC, white blood cell; FAB, French-American-British classification. The cutoff value for *LDLRAD2* was 0.01635, which was defined as the median for all of the AML patients.

Table S2. Demographic and Laboratory Characteristics of Healthy Donors

Donor No.	Sex	Age (years)	Source	Exclusion Criteria	WBC (×10⁹/L)	Hemoglobin (g/L)	Platelets (×10⁹/L)	Karyotype	Gene Mutation Status
HD-01	Male	34	HC	No hema.	5.5	151	236	Normal	Negative
HD-02	Male	29	HC	No hema.	4.9	142	287	Normal	Negative
HD-03	Female	41	HC	No hema.	6.1	138	272	Normal	Negative
HD-04	Male	27	HC	No hema.	5.2	147	309	Normal	Negative
HD-05	Female	32	HC	No hema.	4.7	119	192	Normal	Negative
HD-06	Female	38	HC	No hema.	6.9	131	254	Normal	Negative
HD-07	Male	45	HC	No hema.	9.8	156	285	Normal	Negative
HD-08	Female	31	HC	No hema.	7.2	125	263	Normal	Negative
HD-09	Female	36	HC	No hema.	3.8	134	296	Normal	Negative
HD-10	Male	30	HC	No hema.	5.7	148	227	Normal	Negative
Median (Range)	-	34.3 (29-45)	-	-	5.98 (3.8-9.8)	139.1 (119-156)	262.1 (192-309)	-	-

Abbreviations: HD, healthy donor; WBC, white blood cell.

Uncropped western blot images with molecular weight markers

Fig. 1

F

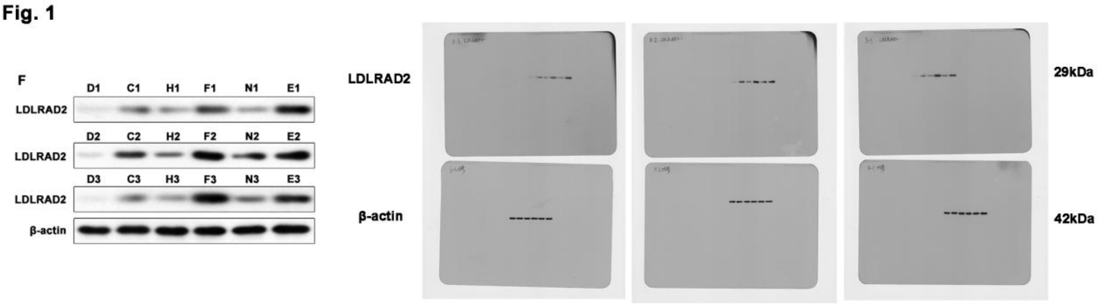

H

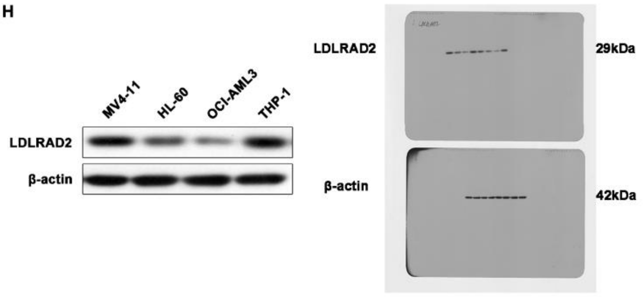

Fig. 3Q

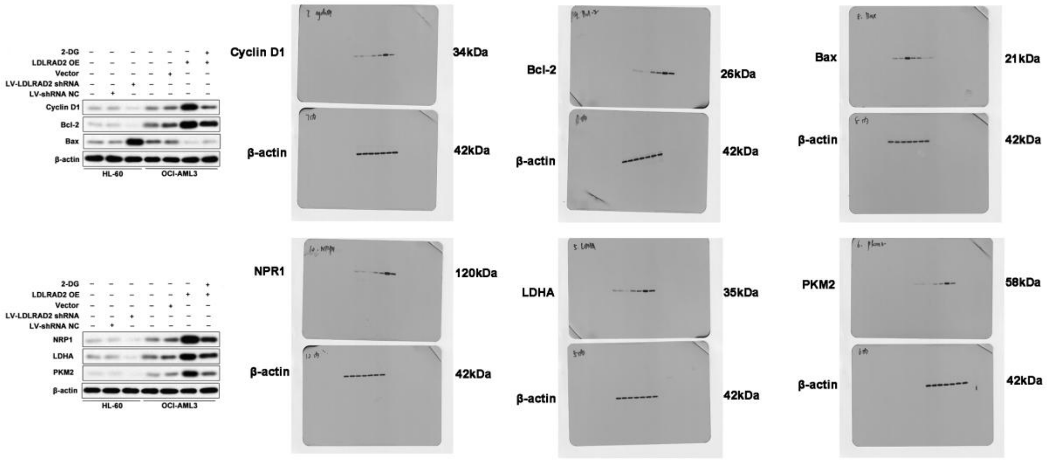

Fig. 5E-5F

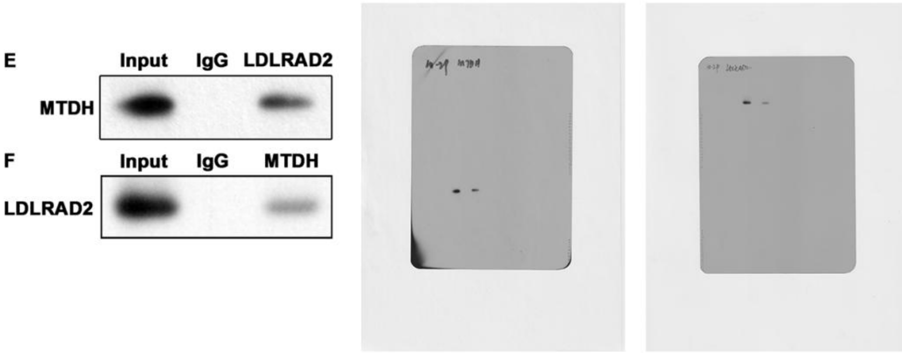

Fig. 5G

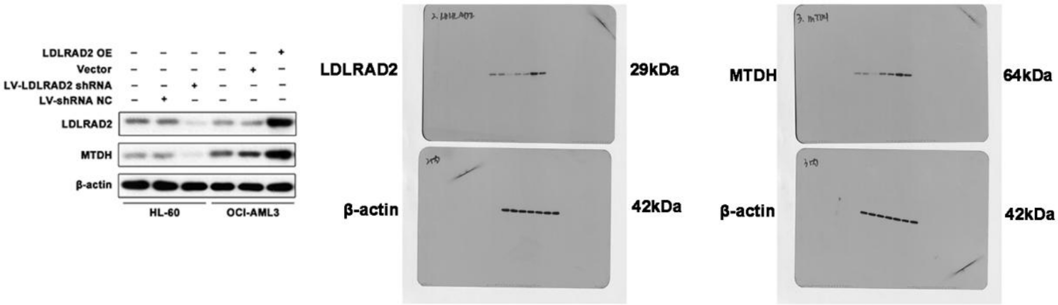

Fig. 5H

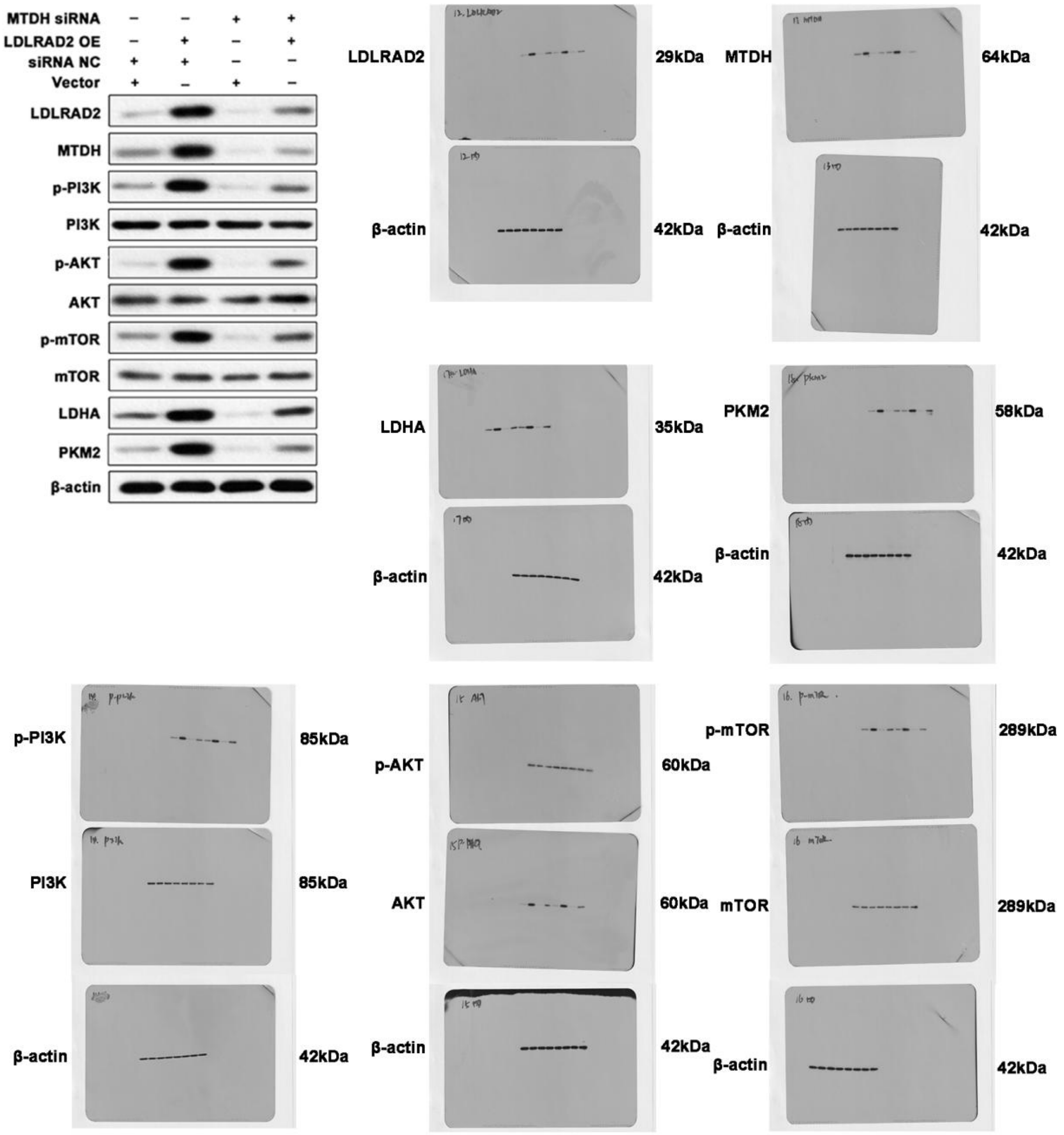
MTDH siRNA − − + +
LDLRAD2 OE − + − +
siRNA NC + + − −
Vector + − + −
LDLRAD2
MTDH
p-PI3K
PI3K
p-AKT
AKT
p-mTOR
mTOR
LDHA
PKM2
β-actin
LDLRAD2
29kDa
β-actin
42kDa
MTDH
64kDa
β-actin
42kDa
LDHA
35kDa
β-actin
42kDa
PKM2
58kDa
β-actin
42kDa
p-PI3K
85kDa
PI3K
85kDa
β-actin
42kDa
p-AKT
60kDa
AKT
60kDa
β-actin
42kDa
p-mTOR
289kDa
mTOR
289kDa
β-actin
42kDa

Fig. 7E

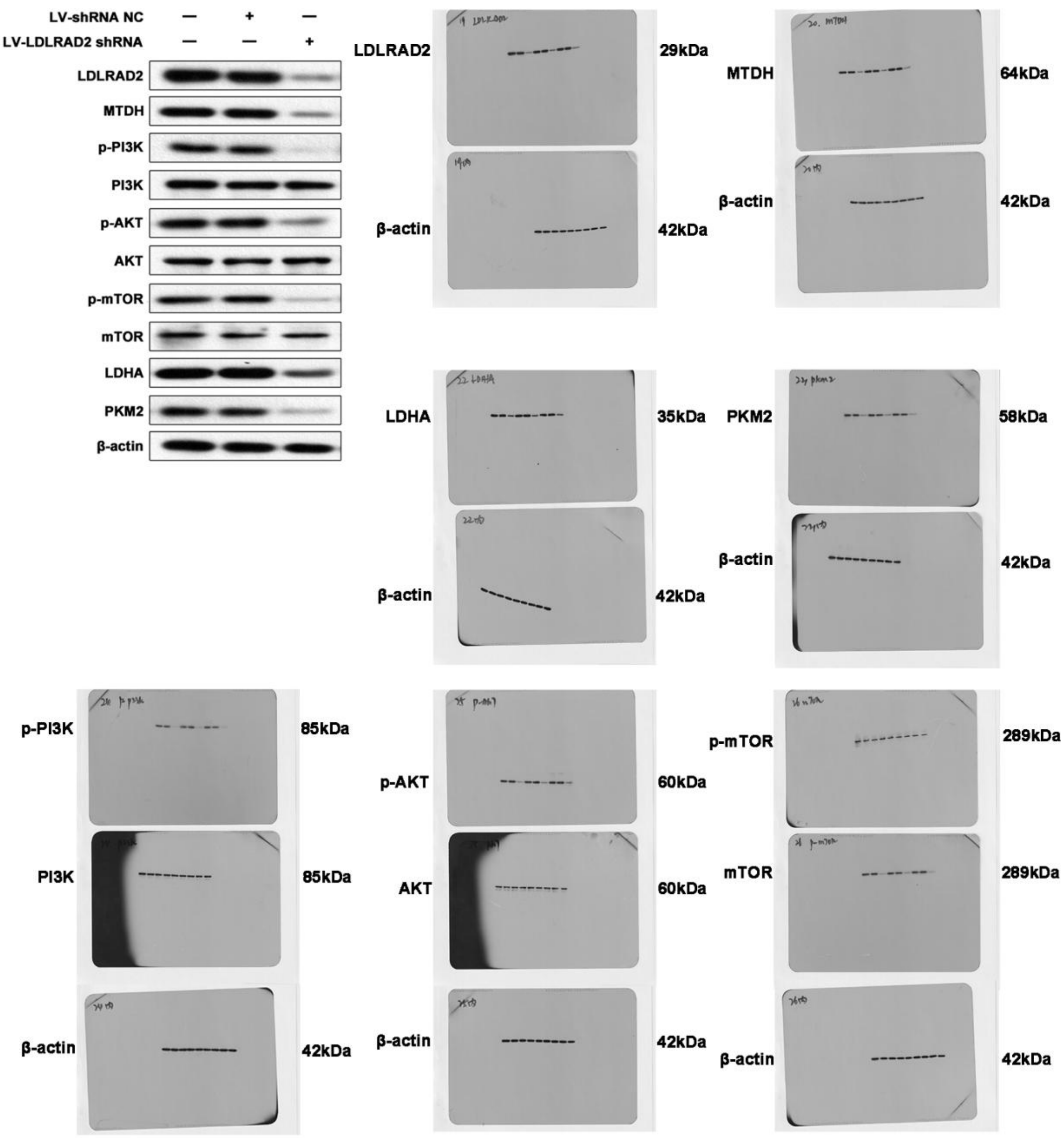
LV-shRNA NC — + —
LV-LDLRAD2 shRNA — — +
LDLRAD2
MTDH
p-PI3K
PI3K
p-AKT
AKT
p-mTOR
mTOR
LDHA
PKM2
β-actin
LDLRAD2 29kDa
β-actin 42kDa
MTDH 64kDa
β-actin 42kDa
LDHA 35kDa
β-actin 42kDa
PKM2 58kDa
β-actin 42kDa
p-PI3K 85kDa
PI3K 85kDa
β-actin 42kDa
p-AKT 60kDa
AKT 60kDa
β-actin 42kDa
p-mTOR 289kDa
mTOR 289kDa
β-actin 42kDa

Fig. S1

C

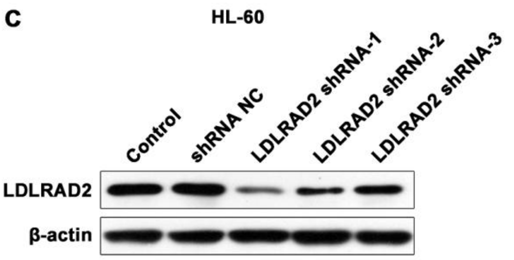

HL-60
Control
shRNA NC
LDLRAD2 shRNA-1
LDLRAD2 shRNA-2
LDLRAD2 shRNA-3
LDLRAD2
β-actin

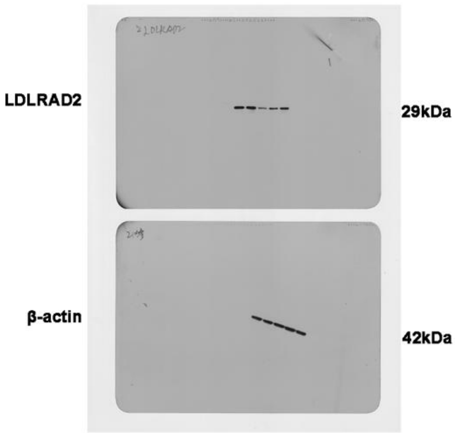

LDLRAD2
29kDa
β-actin
42kDa

F

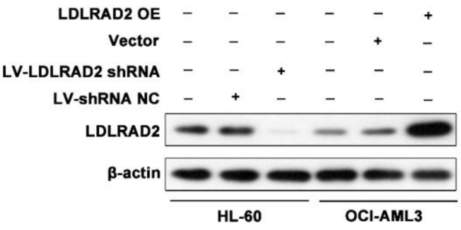

LDLRAD2 OE
Vector
LV-LDLRAD2 shRNA
LV-shRNA NC
LDLRAD2
β-actin
HL-60
OCI-AML3

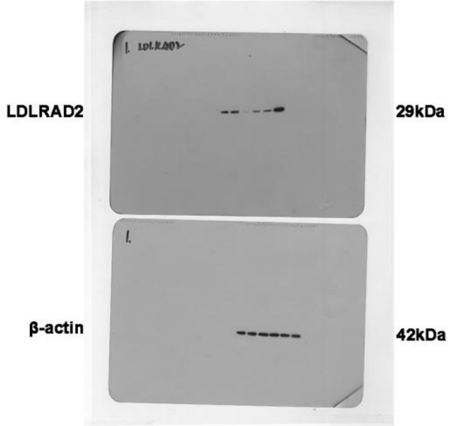

LDLRAD2
29kDa
β-actin
42kDa

I

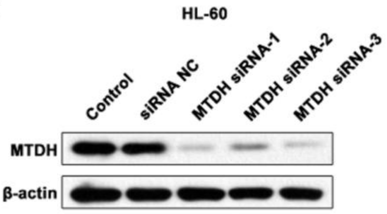

HL-60
Control
siRNA NC
MTDH siRNA-1
MTDH siRNA-2
MTDH siRNA-3
MTDH
β-actin

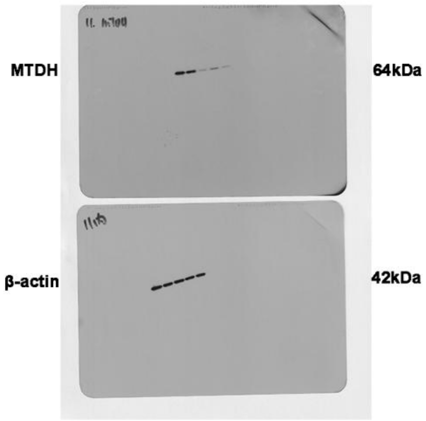

MTDH
64kDa
β-actin
42kDa
